# Role of Natural Autoantibodies and Natural IgM Anti-Leucocyte Autoantibodies in Health and Disease

**DOI:** 10.3389/fimmu.2016.00198

**Published:** 2016-06-06

**Authors:** Peter Isaac Lobo

**Affiliations:** ^1^Department of Internal Medicine, Division of Nephrology, Center of Immunology, Inflammation and Regenerative Medicine, University of Virginia Health Center, Charlottesville, VA, USA

**Keywords:** natural IgG autoantibodies, natural IgM autoantibodies, natural autoantibodies, natural IgM anti-leukocyte antibodies, allograft rejection, TH-17, renal IRI, autoimmune insulitis, regulation of co-stimulatory receptors, regulatory dendritic cells

## Abstract

We review how polyreactive natural IgM autoantibodies (IgM-NAA) protect the host from invading micro-organisms and host neo-antigens that are constantly being produced by oxidation mechanisms and cell apoptosis. Second, we discuss how IgM-NAA and IgM anti-leukocyte antibodies (IgM-ALA) inhibits autoimmune inflammation by anti-idiotypic mechanisms, enhancing removal of apoptotic cells, masking neo-antigens, and regulating the function of dendritic cells (DC) and effector cells. Third, we review how natural IgM prevents autoimmune disorders arising from pathogenic IgG autoantibodies, triggered by genetic mechanisms (e.g., SLE) or micro-organisms, as well as by autoreactive B and T cells that have escaped tolerance mechanisms. Studies in IgM knockout mice have clearly demonstrated that regulatory B and T cells require IgM to effectively regulate inflammation mediated by innate, adaptive, and autoimmune mechanisms. It is, therefore, not surprising why the host positively selects such autoreactive B1 cells that generate IgM-NAA, which are also evolutionarily conserved. Fourth, we show that IgM-ALA levels and their repertoire can vary in normal humans and disease states and this variation may partly explain the observed differences in the inflammatory response after infection, ischemic injury, or after a transplant. We also show how protective IgM-NAA can be rendered pathogenic under non-physiological conditions. We also review IgG-NAA that are more abundant than IgM-NAA in plasma. However, we need to understand if the (Fab)^2^ region of IgG-NAA has physiological relevance in non-disease states, as in plasma, their functional activity is blocked by IgM-NAA having anti-idiotypic activity. Some IgG-NAA are produced by B2 cells that have escaped tolerance mechanisms and we show how such pathogenic IgG-NAA are regulated to prevent autoimmune disease. The Fc region of IgG-NAA can influence inflammation and B cell function *in vivo* by binding to activating and inhibitory FcγR. IgM-NAA has therapeutic potential. Polyclonal IgM infusions can be used to abrogate on-going inflammation. Additionally, inflammation arising after ischemic kidney injury, e.g., during high-risk elective cardiac surgery or after allograft transplantation, can be prevented by pre-emptively infusing polyclonal IgM or DC pretreated *ex vivo* with IgM or by increasing *in vivo* IgM with a vaccine approach. Cell therapy is appealing as less IgM will be required.

## Introduction

The study of natural IgM autoantibodies (IgM-NAA) has given us a good insight on how nature, by creating polyreactive pentavalent antibodies, has accomplished a difficult task of trying to protect the host from both diverse foreign pathogens and from diverse self neo-antigens that are constantly produced within the host. Poly-reactivity with low binding affinity, but with high avidity, has enabled these IgM-NAA-producing B cell clones to be rapidly activated by a foreign or an auto neo-antigen for deploying protective mechanisms to the host. Such a response provides time for the adaptive immune system to mount a highly specific immune response to foreign antigens and, in addition, lessens the burden on the host to maintain diverse B cell clones producing highly specific IgG auto-antibodies, which has the potential of causing autoimmune disease owing to their high-affinity binding. Second, since both foreign and self neo-antigens can induce an inflammatory response, these IgM-NAA have taken over another task of subduing an excessive inflammatory response that can injure the host. This regulation of inflammatory cells without damage is made possible by the low binding affinity of IgM-ALA to live leukocytes, combined with its inability to effectively activate the lytic component of complement at body temperature (37°C). Hence, it is not surprising why these IgM-NAA antibodies, which first arose in cartilaginous fish, have been conserved during evolution [reviewed in Ref. (1)] and why IgM-NAA makes up about 70–80% of circulating IgM (2, 3). Additionally, some studies have shown that the binding of natural IgM to autologous receptors is also evolutionarily conserved among mammalian species as human IgM has the same functional effect as murine IgM on murine cells *in vitro* or when used *in vivo* in mice (4–6).

Natural autoantibodies of different isotypes have been intensively studied during the last 40 years (7–17). These autoantibodies have been termed “natural antibodies” as they are produced at birth in the absence of exposure to foreign antigens. The full repertoire of NAA develops by early childhood. In mice, NAA are predominantly produced by the CD5+ B1 cells, while marginal-zone splenic B (MZB) cells contribute the remainder. These B1 cells produce predominantly IgM, IgA, and IgG3 autoantibodies (18, 19), independently of T cell help, and exhibit an enhanced response to innate immune signals, such as TLR agonist. Hence, B1 and MZB cells differ from B2 cells in that the response of these cells *in vivo* is rapid and can be driven by TLR agonists independently of antigen binding to their BCR (20–23). Additionally, there are data to indicate that autoantibody-producing B1 cells, unlike self-reactive T cells, are positively selected for their self-reactivity, thus implying that NAA are conserved by design (24–27). Further support for their importance comes from studies in chimeric mice demonstrating that IgM-NAA comprise the majority of circulating IgM (2, 3). Most cross-sectional studies in humans and rodents would indicate that IgM-NAA decrease with age (28–31) or lose their effectiveness with age (32) except for one report where follow-up of five healthy individuals for 25 years revealed no change in IgM-NAA levels (33). However, IgG-NAA can increase (34) but do not decrease with age (35, 36).

Innately produced natural IgM-NAA should not be confused with immune IgM and IgG that are produced several days later after exposure to foreign antigens or pathogens. Such immune IgM and IgG are not natural autoantibodies and in general are antigen specific and are produced by B2 cells that require antigen binding to BcR and additional T cell help to generate anti-protein antibodies. However, production of immune IgM is limited as these IgM secreting B2 cells migrate to B cell follicles, where with the help of T cells, these B2 cells undergo isotype switching and somatic hyper-mutation, thus generating long-lived memory B cells and differentiating into plasma cells that produce IgG antibodies with high-affinity binding.

The human equivalent of the murine CD5+ B1 subset has been recently identified and characterized. This CD20+ CD43+ CD27+ human B1 subset that can spontaneously secrete antibody represents about 50% of umbilical cord B cells and 15–20% of circulating adult B cells, and is the predominant source of human IgM-NAA (29). In humans, CD5 is not a specific marker of B1 as this marker is expressed by both B1 and B2 cells. Similarly, CD43 and CD27 are not specific markers for human B1 as about 20% of CD43+ CD 27+ B cells have characteristics of pre-plasmablasts that are derived from T-dependent B cells present in germinal centers (37, 38). Human IgM-NAA are also polyreactive and bind similar autoantigens as in mice, including oxidized neo-determinants and leukocyte receptors (4, 39–41).

### IgM-NAA, IgG-NAA, and Pathogenic IgG Autoantibodies

One physiological role of NAA is to protect the host from pathogenic IgG autoantibodies. We will, therefore, briefly describe the biology of natural IgM and IgG-NAA and pathogenic IgG autoantibodies in health and disease and then discuss the different mechanisms used by NAA to counter pathogenic IgG. B1 cells have been shown to secrete IgM, IgA, and IgG3-NAA that are encoded by minimally or non-mutated germ line genes that are enriched for heavy chain variable region rearrangements and with H-L pairings that allow for poly-reactivity (18, 19). NAA generated by B1 cells rarely undergo isotype switching or somatic hyper-mutation to acquire antigen specificity. IgA in the gut is predominantly produced by B1 cells present in the gut lymphoid tissue but there is increasing evidence to show that B2 cells can also participate in gut mucosal immunity and this could account for the observed somatic hyper-mutation and antigen specificity of gut mucosal IgA (42–46).

#### Natural IgM Autoantibodies

In mice, the majority of IgM-NAA are produced by splenic B1 and MZB cells (47–49). B1 and MZB cells, such as memory B cells, express CD27 and spontaneously produce IgM, but IgM production can also be increased by TLR activation [e.g., lipopolysaccharide (LPS)] or via the BCR in response to pathogens. Normal levels of IgM-NAA are present at birth even under germ-free conditions and in nude mice, indicating that IgM-NAA production is not dependent on exposure to foreign antigens, TLR or T cell activation (50). IgM-NAA repertoire is shaped by T-independent antigen activation, especially of MZB cells (51, 52). An important characteristic of these antibodies is their low binding affinity (53). It is possible that the membrane expressed IgM or BCR of B1 and MZB cells producing NAA also exhibit low binding affinity and perhaps this latter characteristic may be involved in preventing autoreactive B1 cells from being deleted or undergoing negative selection. There are data to show that autoreactive B1 cells are positively selected and this process requires both the autoantigen and the relevant BCR (24–27). The need to positively select B1 cells secreting IgM-NAA would indicate that these antibodies have an important physiological role that will be reviewed later.

Natural IgM autoantibodies have been shown to be polyclonal with clones having specificity for some, but not all self-antigens, i.e., certain common epitopes present on phylogenetically conserved self-antigens. Some of these IgM clones with reactivity to self-antigens have been identified, e.g., IgM clones with specificity for leukocyte receptors [IgM anti-leukocyte antibodies (IgM-ALA)] (4, 39), Fc domain of IgG (rheumatoid factor) (14, 15), complement components (17), collagen, thyroglobulin, intracellular constituents, such as cytoskeletal proteins, cytosolic enzymes, dsDNA, or nucleosomes, neutrophil cytoplasmic enzymes (ANCA) (50, 54) and oxidized neo-determinants [e.g., phosphorylcholine (PC)] that are exposed when lipids are oxidized or cells undergo apoptosis (55, 56). While some IgM-ALA have mono-reactivity, e.g., to some cytokines, most are polyreactive with each polyreactive IgM-NAA clone having a selective binding profile (50). For example, IgM anti-PC NAA will bind to ABO blood type antigens, endotoxins, and oxidized neo-determinants on apoptotic cells but this autoantibody has no binding reactivity to nuclear antigens or to IgG (57). Additionally, these IgM-NAA, by virtue of being polyreactive, also cross-react with pathogen-expressed molecules, for example, PC on *Streptococcus pneumoniae* and other antigens expressed by various viruses and parasites (55–57) Hence, it has been suggested that these natural IgM antibodies are protective, serving as a first line of defense against infections and protecting the host from pathogen-mediated apoptotic cells and oxidized neo-determinants that can induce pathogenic IgG autoantibodies (55, 56). Additionally, polyreactive IgM-NAA have been shown to bind to idiotypic determinants on self-reactive IgG, thus providing another mechanism to protect the host from high-affinity binding IgG autoantibodies that are potentially pathogenic (31, 50).

In mice, B1 cells are rare in the bone-marrow, lymph nodes, and splenic B cell follicles (white pulp) while significant numbers of B1 cells are located in the splenic marginal zone as well as in the peritoneal and pleural cavities. However, under normal conditions, these peritoneal B1 cells do not contribute significantly to circulating IgM-NAA, but during sepsis, peritoneal B1 cells rapidly migrate to the splenic marginal zone (23, 58) where most of the circulating IgM-NAA are produced (59). B1 cells are distinct from B2 cells in many respects and they are derived from different progenitors (18, 19, 60). Importantly B1cells express CD27, a memory B cell marker, and such as memory B cells, on encountering antigen, spontaneously secrete IgM without requiring signaling via co-stimulatory molecules (60). B1 cells do not require to traffic or reside in the splenic B2/T cell follicles as they are T independent and secrete IgM without isotype switching or somatic mutation (25, 59, 61). In fact, isotype switching and somatic hyper-mutation of immunoglobulins in B1 cells is kept in check during an immune response to prevent the development of high affinity, anti-self IgG antibodies (61, 62). In this regard, SPA-1 in B-1 cells inhibits Rap-1 GTP, which enhances somatic hyper-mutation and hence SPA-1 deficient mice generate high-affinity IgG anti-dsDNA and anti-red blood cell autoantibodies and develop lupus nephritis as they age (62). Recent data would indicate that levels of peritoneal B1 and splenic B1 and MZB cells is regulated by serum levels of polyclonal (but not monoclonal) IgM (63–65) that interacts with FcμR expressed by these cells (66) as well as by IgG binding to FcγRIIB (67).

#### IgG-NAA

One can demonstrate the existence of IgG-NAA in normal adult murine or human serum under conditions where serum IgM is either removed or diluted out (31, 68–70), indicating therefore that in serum the functional activity of IgG-NAA is blocked by polyreactive IgM with anti-idiotypic activity (31, 50, 54). There is, however, a large amount of IgG-NAA as significant levels of IgG-NAA can be detected even after diluting serum at 1:500 (34). Both IgG-NAA and IgM-NAA bind to the same phylogenetically conserved self-antigens and 15–20% of normal mouse IgG has been found to be polyreactive (50, 68). IgG produced by B1 cells are characteristically of the IgG3 isotype that have been shown to be polyreactive (18, 19). Clones of B cells secreting IgG4κ with high-affinity binding to dsDNA has been shown to exist in human umbilical cord blood and in the same umbilical cord there were other B cell clones producing IgM antibodies with binding reactivity to idiotypic determinants on IgG4 anti-dsDNA but not to idiotypic determinants on other IgG4 antibodies (34). The above observations would suggest that B cells generating IgG-NAA of different isotypes and affinities are present at birth and the IgG-NAA are rendered functionally inactive by polyreactive IgM with anti-idiotypic activity.

Importantly, IgG-NAA-producing B cells, unlike IgM-NAA-producing B cells, are in an inactive state at birth and in mice these B cells start producing IgG-NAA after exposure to bowel bacteria or foreign antigens (71–73). In humans, it may take more than 2 years before significant levels of IgG-NAA can be detected in the serum (74). It is also possible that autoreactive T cells, which have been shown to exist in normal individuals (75) or infectious agents could activate B cells to produce IgG-NAA later on in life. For example, levels of polyreactive IgG anti-dsDNA have been shown to increase after various infections and these polyreactive IgG anti-dsDNA have been found to cross-react with antigens on micro-organisms, including bacteria (76). It is, therefore, not surprising that low levels of IgG-NAA at birth increase after exposure to infections (71–73).

Studies on IgG-NAA isotypes in normal sera or on the B cell subsets that produce these antibodies are lacking. There are, however, extensive data on an IgG-NAA, i.e., IgG anti-DNA, that is both mono-reactive and polyreactive and present in both normal sera and SLE sera and some of the findings with IgG anti-DNA may apply to other IgG-NAA. Several lines of evidence would favor that B2 cells, with the help of T cells, can generate IgG-NAA. Wardemann et al. clearly demonstrated that 55–75% of early B cell precursors, present in human adult bone-marrow, display auto-reactivity to conserved intracellular cytoplasmic and nuclear constituents. About 80% of these autoreactive B cells are removed at two check points, i.e., centrally in the bone-marrow and in the periphery (77). Second, in human umbilical cord, B cells secreting high-affinity binding IgG4 anti-dsDNA have been isolated and IgG1 anti-myeloperoxidase (ANCA) antibodies have been found in normal human sera, indicating that these high-affinity isotype-switched antibodies are produced by the T cell-dependent B2 cells (34, 78). Furthermore, in the MLR/*lpr* murine model of SLE, IgG anti-dsDNA has been shown to be produced by B2 cells under the influence of excess T helper cells as a result of Fas deficiency (79). Hence, removing T cells ameliorates SLE and reduces production of IgG anti-DNA in this model of SLE (80–82).

Under normal conditions, it is unclear whether the (Fab)^2^ region of IgG-NAA is physiologically active especially since their activity is blocked by poly reactive IgM with anti-idiotypic activity (31, 50). Hence, most of the physiological functions of IgG-NAA have been derived from purified IgG or pooled purified IgG (IVIG). *In vivo* infusion of IVIG has been shown to ameliorate cell-mediated inflammatory processes, e.g., in Kawasaki’s disease or autoantibody-mediated disorders, e.g., in idiopathic thrombocytopenic purpura, anti-factor VIII autoimmune disease, or myasthenia gravis [reviewed in Ref. (83)]. One can show, in *in vitro* studies, that the beneficial effects of purified IgG-NAA can be mediated by both the (Fab)^2^ region and the Fc region of IgG-NAA. In these *in vitro* studies, the (Fab)^2^ region has been shown to (i) block function of activating FcγR receptors expressed by monocytes/macrophages. Such activating FcγR are involved in phagocytosis of IgG autoantibody complexed to platelets, (ii) block idiotypic determinants on IgG autoantibodies, thus neutralizing these antibodies, e.g., their binding to Factor VIII or platelets, (iii) inhibit function of certain pro-inflammatory cytokines by binding to these cytokines or their receptors, and (iv) provide protection against bacterial infection by binding of IgG-NAA to lectins on bacterial surfaces thus enhancing opsonization (84). Similarly, in *in vitro* studies, the Fc region of IgG-NAA has been shown to inhibit the function of B cells, plasma cells, dendritic cells (DC), macrophages, and neutrophils by binding to the inhibitory FcγRIIB receptor expressed by these cells. Activation of FcγRIIB by Fc region of IgG-NAA inhibits production of autoantibodies by B cells and plasma cells. Additionally IVIG ameliorates cell-mediated inflammation, e.g., in Kawasaki’s disease, by activating FcγRIIB and inhibiting the function of dendritic cells, macrophages, and neutrophils.

IgG with anti-idiotypic activity have been found in pathological conditions especially when there is minimal or no IgM with anti-idiotypic activity (85) and increased levels of *in vivo* anti-idiotypic IgG has been associated with amelioration of disease activity mediated by pathogenic IgG autoantibodies, thus indicating that under pathological conditions, *in vivo* IgG with anti-idiotypic activity is functionally active (85, 86). There are data to show that normal levels of IgG autoantibodies, e.g., to myeloperoxidase (MPO) increase >10-fold during disease states (87) and it is possible that under pathological conditions when IgM-NAA with anti-idiotypic activity is low, there is initially a compensatory increase in IgG antibodies with anti-idiotypic activity and the disease does not manifest clinically (85). However, with time, the data would also indicate that there is a decrease in the protective IgM/IgG anti-idiotypic antibodies and the disease manifests clinically (85). Hence, administering IVIG, which is known to have anti-idiotypic activity, could be beneficial in this situation especially if levels of both IgG and IgM-NAA are relatively low.

#### Pathogenic IgG Autoantibodies

Pathogenic autoantibodies that cause disease differ from IgM and IgG3-NAA in that they are predominantly of the IgG isotype, undergo somatic mutation with addition of N regions and these IgG autoantibodies can be either polyreactive or exhibit mono-reactivity. IgG autoantibodies, unlike IgM-NAA, have high-binding affinity (53). However, not all IgG autoantibodies with high-binding affinity are pathogenic *in vivo* and this is best exemplified by IgG anti-dsDNA autoantibodies (88–91). There are conflicting data on the B cell precursors that generate these pathogenic IgG autoantibodies. Data would indicate that both B1 and B2 cells can generate pathogenic IgG autoantibodies depending on the animal model used. For example, studies in the Fas-deficient MRL-*lpr* murine SLE model would indicate that pathogenic self-reactive IgG antibodies are generated by B2 cells (79). However, in the NZB/W disease model of SLE and in a murine transgenic model of hemolytic anemia, the pathogenic murine IgG anti-dsDNA and anti-erythrocyte transgenic autoantibodies were shown to be produced by B1 lymphocytes (21, 92–94). Hence, removing T cells did not affect production of IgG anti-dsDNA or disease activity in the NZB/W model of SLE (80).

There are several mechanisms that increase pathogenic IgG antibody production and IgG anti-dsDNA has been most studied in this regard. In adolescent females with SLE and rheumatoid arthritis (RA) patients, there are data to show that production of IgG polyreactive autoantibodies results from breakdown of B cell tolerance mechanisms where during B cell development, B cells producing high-affinity IgG autoantibodies have not been removed or silenced either centrally in the bone-marrow or in the periphery before they mature into naïve immuno-competent lymphocytes (95, 96). Studies in healthy individuals indicate that 55–70% of newly generated bone-marrow B cells express self-reactive antibodies and most of these B cells are removed or silenced such that in healthy humans there remains 5–20% of circulating naïve B cells that continue to generate self-reactive IgG autoantibodies (77). However, in SLE and RA patients, owing to breakdown of B cell tolerance mechanisms, 25–50% of mature naïve B cells were found to generate self-reactive IgG autoantibodies with diverse specificities (95, 96). Second, excess BAFF could also contribute to the breakdown of tolerance in the periphery as BAFF has been shown to inhibit deletion or apoptosis of self-reactive B cells (92, 97). Elevated BAFF levels have been shown to be present in serum of RA and Sjogren’s syndrome (98). Third, there are murine studies to show that IgM-NAA-producing B1 cells can be induced, especially with repeated immunization, to generate polyreactive IgG anti-self that can also react to the immunizing antigen. For example, B1 cells can be induced to generate pathogenic polyreactive IgG anti-DNA and anti-myosin antibodies by immunization with a hapten or an antigen derived from a micro-organism (99–101). Other mechanisms may, however, operate in certain other autoimmune disorders where highly specific IgG autoantibodies are produced, e.g., in autoimmune hemolytic anemia and in Factor VIII deficiency. It is possible that such autoimmune disorders could arise from a breakdown in peripheral mechanisms such as specific deficiency of anti-idiotypic IgM-NAA that blocks pathogenic autoantibody or specific deficiency of IgM-NAA that binds and masks autologous neo-determinants such as dsDNA.

Based on observations in the preceding paragraph, it appears that different mechanisms could operate in generating pathogenic IgG autoantibodies depending on the murine disease model. Additionally, the mechanisms may differ in humans even though the disease has similar disease manifestations. A summary of the existing evidence would indicate that IgG autoantibodies can be induced by the following mechanisms:

(i) An excessive increase in production of pathogenic IgG autoantibodies relative to IgM-NAA-producing B1 cells. Potential mechanisms that increase self-reactive IgG antibodies include lack of helper T cell apoptosis [e.g., in Fas-deficient MRL-*lpr* mice (79)], excess neoantigen-driven B cells [e.g., in human C1q deficiency with decreased removal of apoptotic cells (102)], excess BAFF production [e.g., in patients with Sjogren’s syndrome (98)], a breakdown in B cell tolerance mechanisms resulting in excess naïve self-reactive B cells [e.g., in SLE affecting young female patients (95)] and a deficiency in the inhibitory FcγRIIB receptor that normally inhibits B cells and plasma cells from producing IgG antibodies (103, 104).
(ii) Genetic predisposition that permits some antigen-driven B1 cell clones to acquire the ability for isotype switching and somatic mutation as, for example, in the NZB/W murine lupus model and in SPA-1 deficient mice (105, 106, 62). Hence, diseases induced by such defective B1 cells are ameliorated with administration of either polyclonal IgM or the specific IgM-NAA (107) or depleting B1 cells (94).
(iii) Repeated immunization that induces isotype switching in B1 cells that normally generate IgM autoantibodies with germline genes. For example, repeated immunization with hapten or a streptococcal antigen can induce B1 cells to generate IgG antibodies of different isotypes that are polyreactive and bind to the immunizing antigen as well as to self-antigens, such as DNA or myosin (99–101).
(iv) Excess production of a highly specific pathogenic IgG autoantibody by autoreactive B2 cell clones as, for example, in myasthenia gravis (108). Expansion of such B2 cell clones could have been driven by antigen, e.g., with cross-reactive micro-organisms in patients without thymoma (109) or be driven by acetylcholine receptor specific autoreactive helper CD4^+^ T cells in thymoma-associated myasthenia gravis where such autoreactive T cells have escaped tolerance mechanisms (110, 111).
(v) Deficiency of IgM-NAA, especially IgM-NAA with anti-idiotypic activity to a specific IgG autoantibody, thus incompletely neutralizing the increase in pathogenic IgG autoantibodies, such as antibodies to neutrophil cytoplasmic enzymes (myeloperoxidase, proteinase 3), which are commonly referred to as ANCA, dsDNA, and glomerular basement membrane (GBM) that are present in normal individuals (31, 50, 87, 112). Additionally, certain autoimmune disorders, such as ANCA vasculitis and anti-GBM nephritis, frequently occur in elderly patients who, with aging, can develop lower levels of IgM-NAA (28–32) but not IgG-NAA (33, 35, 36), which can increase with aging (34).

**Figure 1:**
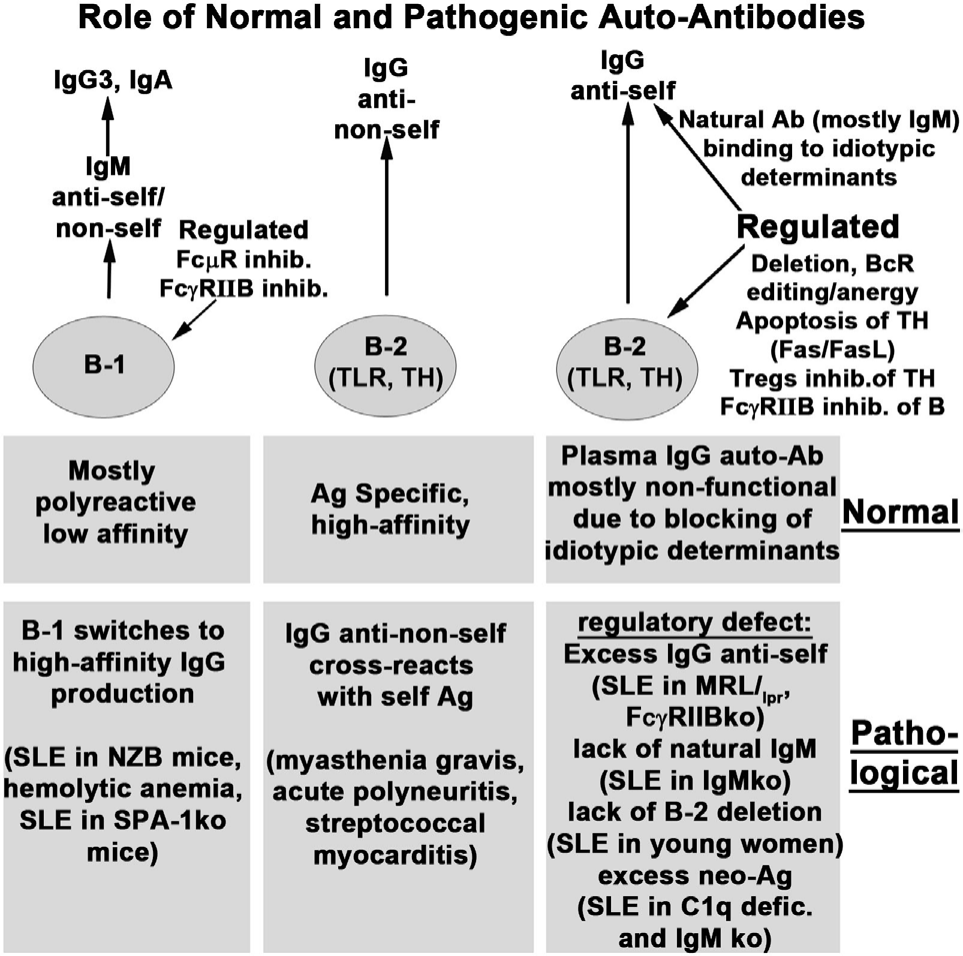
**Role of normal and pathogenic autoantibodies**.

Figure 1 summarizes the role of natural and immune antibodies in normal and pathological states.

#### Role of IgM-NAA in Protecting Against Pathogenic IgG Autoantibodies

SLE is the most studied autoimmune disorder where high levels of IgM and IgG autoantibodies, directed against nuclear antigens, especially dsDNA, are produced in the majority of patients. Before we discuss the role of IgM-NAA in protecting against pathogenic IgG anti-dsDNA, it would be important to indicate that a subset of lupus nephritis (approximately 25%) can occur in the absence of detectable circulating IgG anti-DNA and this is commonly referred to as C1q nephropathy (113, 114). The latter observations have led some investigators to question whether IgG anti-dsDNA is indeed pathogenic (115). In some patients with C1q nephropathy, circulating IgG anti-dsDNA can be detected several years later but this subset of patients with C1q nephropathy have in general a better prognosis even though histologically there is similar glomerular immunoglobulin staining and immune deposits as in classical lupus nephritis (114). It is tempting to speculate that patients with C1q nephropathy may have high IgM-NAA and IgM anti-dsDNA that may be preventing detection of circulating IgG anti-dsDNA. A murine model with lupus nephritis but with no IgG anti-dsDNA in both the circulation and in kidney eluates has also been described (116). It is possible that in this murine model, the immune deposits detected in the kidney may be caused by IgG complexes containing another antigen, other than dsDNA, and a similar mechanism could also operate in some patients with C1q nephropathy without circulating IgG anti-dsDNA.

However, there is evidence indicating that IgG anti-dsDNA, together with C1q, is an active participant in inducing or initiating glomerular damage in classical lupus nephritis with circulating IgG anti-dsDNA. First, SLE in the MRL*/lpr* lupus-prone mice is ameliorated when rendered IgG deficient by crossing these mice with either activation-induced deaminase (AID) or CD40L-deficient mice (117, 118). Second, one can induce lupus nephritis in normal mice by either infusing purified IgG anti-dsDNA or by increasing *in vivo* IgG anti-dsDNA with use of transgenic cells or hybridoma cells (88–91). Importantly, not all IgG anti-dsDNA are pathogenic as defined by induction of lupus nephritis *in vivo* and this also may explain why certain patients with high levels of circulating IgG anti-DNA are spared from lupus nephritis (88–91). In SLE, a potential protective mechanism involves binding of IgM anti-dsDNA to DNA with masking of the antigenic determinants that bind to IgG anti-dsDNA. Hence, lupus nephritis can occur if there is excess IgG anti-dsDNA or if there is a relative deficiency of IgM anti-dsDNA. Such a concept will explain the accelerated development of lupus nephritis in MRL*/lpr* mice lacking IgM and will also explain the lack of lupus nephritis in patients having high IgG anti-dsDNA and high IgM anti-dsDNA or IgM-NAA (119, 120). This latter possibility may also explain why FcγRIIB-deficient Balb/c mice are more resistant to developing lupus nephritis when compared to FcγRIIB-deficient C57BL/6 mice (121). In both mice, there is excess IgG anti-dsDNA production due to lack of the inhibitory FcγRIIB receptor but Balb/c mice have significantly higher levels of plasma IgM when compared to C57BL/6 mice (122–124).

Several observations would suggest a role of IgM-NAA in protecting normal individuals and patients with autoimmune disorders from such self-reactive IgG antibodies. The protective role of IgM-NAA is best exemplified in murine models of SLE, where enhancing IgM anti-dsDNA levels or infusing IgM anti-dsDNA, ameliorates disease activity (107, 125, 126). Well studied mechanisms by which IgM-NAA can induce protection and prevent inflammation include (i) removing neo-antigens by binding of IgM-NAA to oxidized neo-determinants and removal of IgM/C1q bound antigens by phagocytosis, e.g., IgM anti-PC (127), (ii) inhibition of the pathogenic IgG autoantibody induced inflammatory response by IgM-NAA, e.g., IgM-ALA (128), (iii) neutralization of pathogenic IgG autoantibodies by IgM-NAA having anti-idiotypic activity (129), (iv) preventing binding of IgG to antigenic determinants by competitive inhibition even though these IgM-NAA have lower binding affinity (129), (v) inhibiting complement activity by binding of IgM to complement components (17), and (vi) inhibiting generation of IgG autoantibodies possibly by binding of IgM-NAA to endogenous autoantigens/neo-antigens or to the B cell FcμR (64, 65, 119, 130–132).

The above mechanisms may explain why mice without secretory IgM have increased IgG autoantibodies, including anti-dsDNA, with aging (119, 120). This would also explain why B1-deficient SJL mice are more susceptible to induction of experimental autoimmune thyroiditis or allergic encephalitis cells (133). Conversely, such a concept would also explain why disease activity in SLE patients is less severe with high levels of IgM anti-dsDNA and other IgM-NAA (125, 134, 135). The above mechanisms may also explain why several autoimmune disorders are helped by either increasing production of *in vivo* IgM-NAA (126) or administering either pooled polyclonal IgM or a specific IgM-NAA (110, 129, 136). The latter is best exemplified by IgM anti-dsDNA where infusion of a specific IgM-NAA ameliorated SLE-induced nephritis in NZB mice (107).

Presence of pathogenic IgG autoantibodies leads to a compensatory increase in IgM–NAA, such as IgM anti-PC, IgM-ALA, and IgM/IgG anti-idiotypic antibodies, to counter these pathogenic IgG antibodies as well as inflammation initiated by the excess IgG self-reactive antibody. It is also possible, that over time, the production of IgM and IgG anti-idiotypic antibodies may not be able to keep up with the relatively high production of pathogenic IgG autoantibody and the disease becomes symptomatic. Hence, the initial increase in IgM-NAA and IgG anti-idiotypic antibody levels could explain the asymptomatic nature of the disease despite increased levels of IgG anti-dsDNA and other anti-nuclear antibodies several years before clinical onset of disease (85, 137). Additionally, there could be a heightened compensatory response of other immuno-regulatory mechanisms, e.g., increase in T-regs or IL-10 in B1 cells, which could inhibit the clinical onset of disease or ameliorate disease activity in these patients and other animal models. The latter is best exemplified in mice with secretory IgM deficiency, where despite total lack of circulating IgM and increased IgG3 anti-dsDNA (120), these cellular compensatory mechanisms inhibit development of autoimmune disease including lupus nephritis (63, 119). It is also possible that IgG3 anti-DNA is not pathogenic and their increased production by B1 cells, in secretory IgM deficiency (120), may be protective. However, in situations where inflammation has been experimentally induced or there is an excessive increase in production of pathogenic IgG autoantibodies or increased apoptosis, then these cellular regulatory mechanisms cannot effectively compensate for lack of IgM and this leads to a more severe and rapid worsening in disease activity. For example, SLE disease is more severe in the MRL-*lpr* mice when these mice are crossed with secretory IgM deficient mice (120). Similarly, secretory IgM-deficient mice are prone to develop a more rapid and severe inflammatory response after renal ischemia or allogeneic cardiac transplantation (138).

### Physiological Role of IgM-NAA

**Figure 2:**
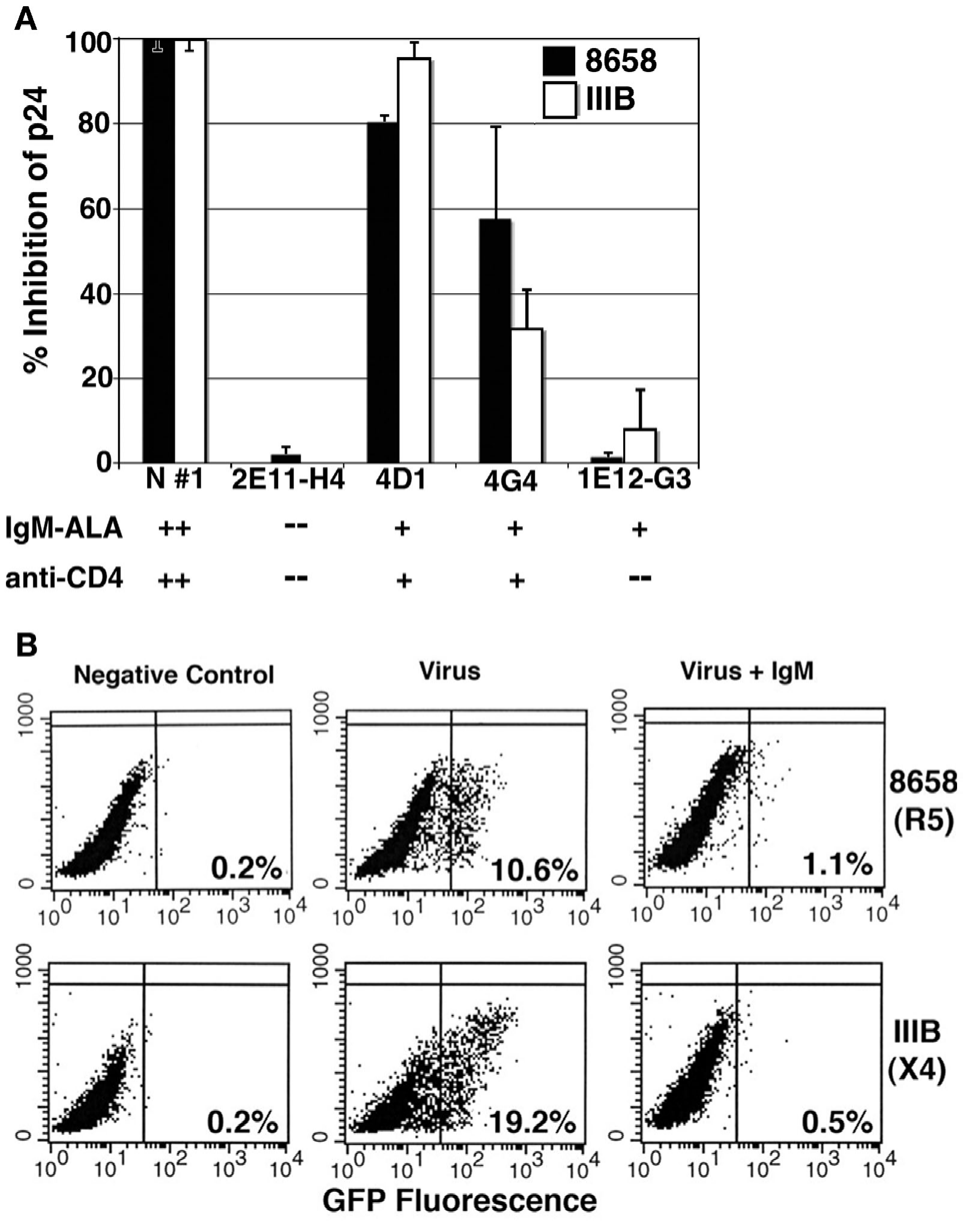
**Evaluating the inhibitory effect of human monoclonal IgM and polyclonal IgM on R5-8658 and X4-IIIB HIV viruses**. **(A)** Specificity of umbilical cord B cell clone IgM was characterized by binding of IgM, to recombinant CD4 and to leukocytes, which is depicted below each clone. IgM from B cell clones was used at concentration of 3 μg/ml, while purified IgM from normal serum (positive control) was used at 10 μg/ml. B cell clone 2E11-H4 with no IgM-ALA and no anti-CD4 activity was used as a negative control. Without IgM, p-24 levels for R5-8658 and X4-IIIB were 87,500 and 126,000 pg/ml, respectively. These data are representative of three different experiments. **(B)** The inhibitory effect of normal polyclonal IgM (5 μg/ml) on HIV-1 infection (single cycle replication) of GHOST cells that express GFP upon integration on viral HIV-1 DNA. IgM was present throughout the culture period and added to cells 15 min before adding virus. These are representative examples of five separate experiments. Figure and legend reproduced with permission from Ref. (144). Copyright 2008. The American Association of Immunologists, Inc.

**Figure 3:**
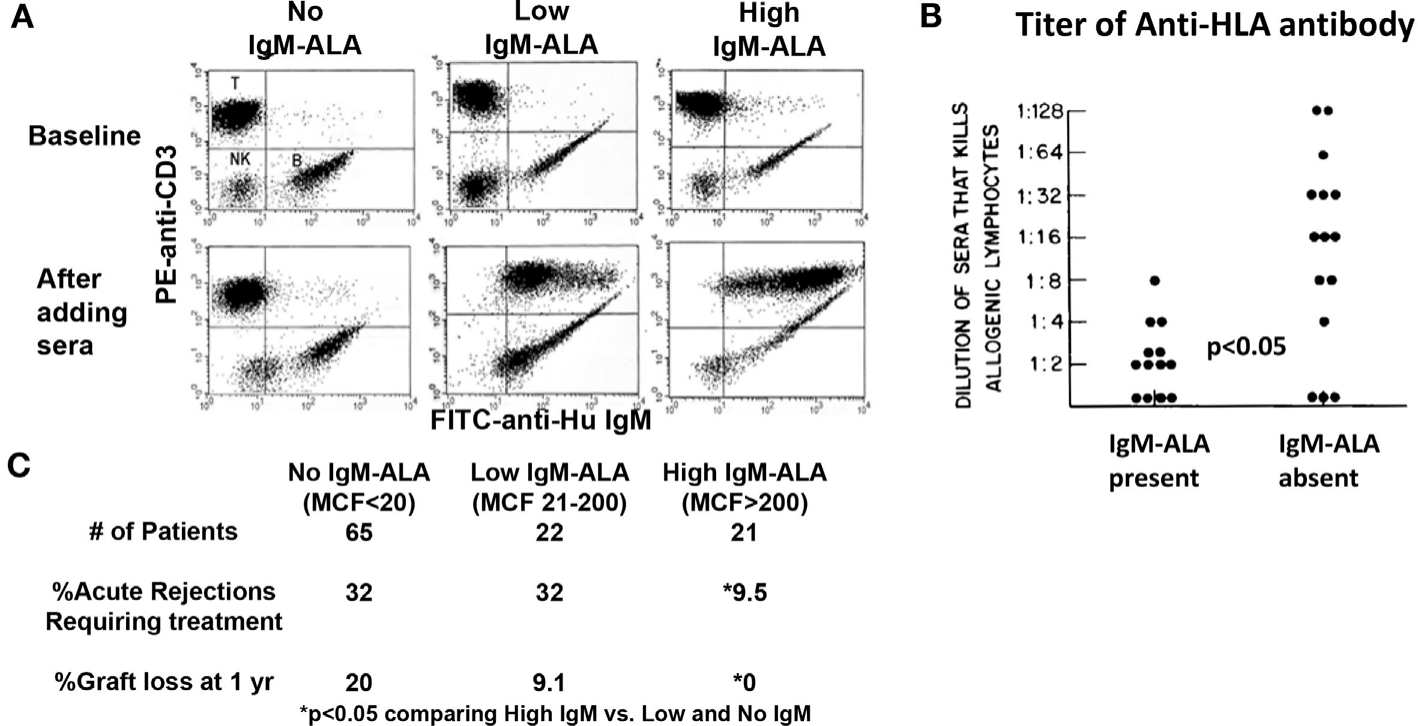
**High levels of serum IgM-ALA in transplant recipients are associated with better kidney allograft survival and decreased alloantibody levels**. **(A)** Dot plots labeled “baseline” depict IgM staining on B lymphocytes, but not on T cells, in the absence of sera. The *lower panels* (after adding sera) depict differences in the level of IgM bound to donor T lymphocytes (IgM-ALA) after addition of pre-transplant serum from different ESRD patients. **(B)** Alloantibody titer in sera measured by cytotoxicity is correlated to presence of IgM autoantibody to leukocytes. **(C)** The difference in percentage of acute rejections and graft loss comparing high IgM-ALA vs. the no and low IgM-ALA groups. *MCF* indicates increase in mean channel fluorescence of anti-IgM staining after addition of serum. Figure and legend reproduced with permission from Ref. (131) copyright 1981 Wolters Kluwer Health Inc. **(B)** Figure and legend reproduced with permission from Ref. (158). Copyright 2010 Springer Science + Business Media LLC **(A,C)**.

IgM–NAA, which are spontaneously secreted by B1 and MZB cells, are polyclonal and exhibit a diverse repertoire of antigen specificities, many of which are polyreactive and, hence, cross-react with foreign antigens or pathogens. Additionally, B1 and MZB cell clones can be activated either by their TLR or BCR by both endogenous and foreign antigens. These special characteristics of IgM-NAA, together with the low-binding affinity (but with high-binding avidity owing to its pentameric structure), have allowed these antibodies to acquire functions that are not characteristic of the highly specific immune IgM and IgG antibodies. Physiological functions that have been attributed to IgM-NAA have included the following:

(i) *Countering the pathogenic effects of the normally present IgG autoantibodies that escape B cell tolerance mechanisms*. Potential strategies employed by IgM-NAA for this purpose include (a) neutralization of these IgG autoantibodies by anti-idiotypic mechanisms (129), (b) masking endogenous antigens (129), and (c) inhibiting the expansion of B2 cell clones that produce these IgG autoantibodies (79, 126). We have discussed these mechanisms in an earlier section of this review. However, the most compelling data demonstrating that IgM-NAA can counter pathogenic IgG autoantibodies are studies in murine models of SLE where administration of IgM anti-DNA or increasing expression of IgM anti-DNA in MRL-*lpr* mice (by crossing MRL-*lpr* mice with transgenic mice expressing IgM anti-DNA) ameliorated disease activity (107, 126).
(ii) *Providing the first line of defense against pathogens while the adaptive immune system*, i.e., *B2 and T cells, are being deployed to mediate a more specific and effective immune response that is long lasting and has memory*. The role of NAA in mediating protection against pathogens was first brought to light by the observation that NAA recognize bacterial toxins and several viral antigens (139, 140). Second, the creation of mice deficient in secretory IgM clearly aided in analyzing the protective role of both natural and immune IgM (63, 141). This topic will be briefly discussed as there are several excellent and detailed reviews on this subject (55, 141). Briefly, IgM-NAA mediates this protection through several mechanisms. First, each natural IgM clone, by having a pentavalent structure and by being polyreactive, can simultaneously bind to different conserved structures, such as nucleic acids, phospholipids, and carbohydrates, on the same pathogen and inhibit the pathogen from invading cells and disseminating into different organs. Second, the early components of complement, such as C1q, bind to the IgM complexed to the invading organism. Complement binding to IgM not only enhances neutralization of pathogens, e.g., certain viruses and bacteria, but also enhances phagocytosis of pathogens by macrophages and dendritic cells. The importance of either natural or immune IgM in limiting infection varies with different pathogens and this subject has been well reviewed (55, 141). Third, normally present IgM-NAA has significant levels of IgM anti–PC, which can readily bind to PC that is attached to sugar residues on the cell membranes and cell walls of many invading organisms (142). PC was first detected in 1967 on *S. pneumoniae* (143) and subsequently found to be present on many bacteria, protozoa, fungi, and nematodes (142). Infection with many of these organisms increases levels of IgM anti-PC, which in the presence of C1q enhances phagocytosis of these organisms and inhibits an excess inflammatory response by mechanisms discussed below. Finally, we have shown that IgM-NAA can limit HIV infection, both *in vitro* and in humanized SCID mice through another mechanism, that is by inhibiting T cell activation (39) as well as by binding of natural IgM to CD4 and chemokine receptors and inhibiting HIV entry into cells (Figure 2) (39, 144).
(iii) *Inhibiting IgG autoantibody production and inflammatory responses by clearing apoptotic cells and binding to oxidized neo-determinants*. Apoptotic cells that are normally produced in large numbers, express oxidized neo-determinants. Several homeostatic mechanisms, including binding of C1q and mannose binding lectin (MBL) to apoptotic cells, are involved in inducing macrophages and DC to phagocytose apoptotic cells and nuclear antigens [reviewed in Ref. (145)]. IgM-NAA are also involved in enhancing this phagocytic process, especially after tissue injury of nerves (69), and in inducing an anti-inflammatory effect (56). Several IgM-NAA antibodies have been identified, each with binding specificity to certain oxidized neo-determinants, including PC, malondialdehyde (MDA), cardiolipin, phosphatidylserine, and Annexin IV present on apoptotic cells and not live cells (146). The best-studied IgM-NAA reactive to apoptotic cells is the IgM anti-PC antibody (also referred to as T-15), which binds to a dominant oxidized neo-determinant on apoptotic cells. Both *in vitro* and *in vivo* studies, using monoclonal IgM anti-PC, have clearly shown that phagocytosis by DC and macrophage is enhanced when C1q or MBL binds to the IgM/apoptotic cell complex. In these *in vivo* studies, either endogenous production of IgM anti-PC was increased by administering large numbers of apoptotic thymocytes (2.5 × 10^7^) or mice were given 1.5–2 mg of a monoclonal IgM anti-PC preparation that bound to endogenous apoptotic cells. Hence, natural IgM anti-PC inhibits production of pathogenic IgG autoantibodies by masking oxidized neo-determinants and enhancing removal of apoptotic cells. However, enhancing apoptotic cell phagocytosis by DC also inhibits DC maturation/activation and induces an anti-inflammatory effect with decreased production of pro-inflammatory cytokines and an increase in levels of splenic regulatory B cells (127, 147). Additionally, repeated weekly infusions of monoclonal IgM anti-PC or apoptotic thymocytes inhibited development of arthritis and induced regulatory B cells in mice (127, 147).
  Natural IgM autoantibodies has also been shown to bind to various oxidation-specific neo-determinants, specifically PC and MDA present on oxidized LDL lipids, but not native LDL (146, 148, 149). High levels of IgM anti-PC and IgM anti-MDA were also found in ApoE-deficient mice that have high circulating cholesterol and severe atherosclerosis. It has been suggested that oxidized lipids, in this murine model, induces these high IgM anti-PC/MDA antibodies as a protective mechanism. Supporting such a concept are other observations showing an association of high *in vivo* levels of IgM anti-PC and reduced atherosclerotic plaques in both patients and different animal models [reviewed in Ref. (56)]. Similarly, there are studies showing that immunization of LDL receptor knockout mice with the *S. pneumoniae* vaccine induces IgM anti-PC and this is associated with less atherosclerotic lesions (150). Several mechanisms have been postulated to explain the amelioration of atherosclerotic lesions by IgM anti-PC in these murine models of atherosclerosis. First, IgM anti-PC masks the PC containing neo-determinants on oxidized lipids, thus inhibiting uptake of these lipids by macrophages and this leads to a decrease in foamy macrophages that are part of the atherosclerotic plaque (151). Second, IgM anti-PC, together with C1q, enhances phagocytosis of apoptotic foamy macrophages containing high levels of lipids. Enhanced phagocytosis of apoptotic macrophages, especially by DC, inhibits DC maturation/activation and induces an anti-inflammatory milieu (127, 147). Data demonstrating increased atherosclerosis in secretory IgM-deficient mice or in C1q ko mice would lend support to the role of IgM anti-PC and C1q in inhibiting atherosclerosis (102, 141, 152–154).
  The finding that both IgM-deficient mice and C1q knockout mice have increased apoptotic cells as well as develop SLE would indicate that both IgM anti-PC and C1q are required to effectively remove or clear apoptotic cells (155, 156). The latter studies would also indicate that inefficient clearance of apoptotic cells, including nuclear antigens, induces development of pathogenic IgG autoantibodies and SLE. However, ineffectual clearance of apoptotic cells cannot entirely explain the increased propensity of IgM-deficient mice to develop autoimmunity and more severe atherosclerosis when compared to C1q ko mice as both mice have similar number of apoptotic cells in the atherosclerotic plaques and the C1q ko mice has in addition, circulating apoptotic cells that should have worsened atherosclerosis (102, 141, 155). Such observations would indicate that IgM-NAA regulates inflammation by other additional mechanisms (to be presented below) besides enhancing phagocytosis of apoptotic cells.
(iv) *Inhibiting inflammation by binding of IgM-NAA to receptors on live leukocytes, i.e., via IgM-ALA*. The existence of this IgM-NAA subset was known since 1970 (157). Their role in inflammation was recognized when several investigators showed that the level of these antibodies, like IgM anti-PC, increased with diverse infections and inflammatory states [reviewed in Ref. (39)]. However, it was unclear whether IgM-ALA were pathogenic or not. The idea that IgM-ALA may have anti-inflammatory function and may be protective came from observations in allograft recipients where patients with high levels of IgM-ALA were found to have significantly less rejections and developed less alloantibodies after allo-immunization (Figure 3) (39, 131). We hypothesized that these natural IgM-ALA increase during inflammatory states to regulate leukocyte function and prevent excess inflammation that may be detrimental to the host. Two characteristics inherent in IgM-ALA allowed us to make this hypothesis. First, we showed that these antibodies bind with low affinity to different leukocyte receptors, including co-stimulatory molecules and chemokine receptors (4, 39). Second, we and other investigators observed that these complement fixing antibodies did not lyse leukocytes at body temperature but readily lysed cells at room temperature or colder temperatures in the presence of complement (131, 157, 159). This non-lytic nature of these antibodies at body temperature led us to hypothesize that IgM-ALA, by binding to receptors on live leukocytes at body temperature, could alter or regulate their function. Initially, with *in vitro* studies using human IgM, we showed that the repertoire of IgM-ALA varies among individuals during health and in disease and, second, that IgM-ALA regulates the function of both human and murine T cells and dendritic cells (4, 39, 138). Additionally, in murine models, we showed that increasing *in vivo* levels of IgM-ALA with physiological doses of purified polyclonal IgM, i.e., 150 μg/mouse, every 3 days, protected mice from (a) renal ischemia reperfusion injury (IRI), (b) ameliorated rejection in a fully mis-matched cardiac allograft model, and (c) protected NOD mice from developing autoimmune insulitis and diabetes. In further experiments, we show that the protective effect in the model of innate inflammation (i.e., renal IRI) is mediated in part by IgM-ALA binding to activated/mature DC and regulating their function (4). In a later section, we will review in more detail the above data on IgM-ALA. We plan to show that the observed anti-inflammatory effects of polyclonal IgM was predominantly from IgM-ALA and not from IgM anti-PC that bound to apoptotic cells.
(v) *Inhibiting expansion of B1 cells and enhancing antigen presentation to B2 and helper T cells in splenic lymphoid follicles*. This physiological function of IgM became obvious in mice with secretory IgM deficiency as well as in mice with FcμR deficiency. B1 cells in the splenic marginal zone increased several fold in mice deficient in IgM while levels of IgM increased in FcμR-deficient mice indicating that IgM maintains B1 cell homeostasis and levels of natural IgM by binding to FcμR (63, 64, 66, 160). Second, both these mice have impaired immune IgG response to protein antigens, especially with low dose antigen, indicating that IgM facilitates antigen trafficking from the splenic marginal zone to the B2 cell rich lymphoid follicles via FcμR bound IgM/antigen complexes (63, 160).

The above observations indicate that IgM-NAA protect the host from invading organisms and more importantly maintain several homeostatic mechanisms primarily aimed at preventing autoimmunity and over exuberant inflammation, which can have detrimental effects on the host. Table 1 summarizes some of the physiological and pathological concepts outlined above. Several observations indicate that infective and other inflammatory states increase all IgM-NAA subsets, especially IgM anti-PC to clear the increased production of apoptotic cells that could trigger autoimmunity and, second, increase IgM-ALA to subdue excess inflammation that can be detrimental to the host [reviewed in Ref. (56) and next section]. Based on the preceding observations, one could predict that a decrease in IgM-NAA, as can occur in aging (28–30), could predispose to increased autoimmunity and increased morbidity and mortality from an excess inflammatory response, for example with infections.

**Table 1:** **Physiologic function of non-pathogenic autoantibodies**.

	**IgM natural antibodies**	**IgG natural antibodies**
Protection from micro-organisms	Binds to bacteria and enhances phagocytosis (req. C1q, Fcα/μR)	Binds to bacteria and enhances phagocytosis (req. C1q, FcγRI)
	Inhibits HIV by blocking entry and inactivating cells	
Prevent autoimmunity	Blocks anti-self IgG Ab (anti-idiotypic)	Blocks anti-self IgG Ab (anti-idiotypic)
	IgM masks neo-antigen	Binds to nuclear and cytoplasmic debris and enhances phagocytosis (req. C1q, MBP)
	Binds to apoptotic cells (PC), nuclear and cytoplasmic debris and enhances phagocytosis (req. C1q, MBP)	
Abrogate inflammation	Anti-complement	Regulates B cells, plasma cells, DC, macrophages, and neutrophils via FcγRIIB
	IgM-ALA ↓ iTNF-α, IL-17, IFN-γ	
	IgM-ALA regulates DC and T cells, and enhances Tregs by binding to CD40, CD86, CD4, CD3, and TcR	
	IgM-ALA blocks chemokine receptors	
B cell homeostasis	IgM regulates B1 cell expansions via FcμR	IgG regulates B1 and B2 cell expansion via FcγRIIB

### Pathogenic Effects of IgM-NAA Under Non-Physiological Conditions

Certain IgM-NAA which under physiological conditions are protective and anti-inflammatory can under non-physiological conditions become pathogenic and induce inflammation. In this section, we will present some of the conditions that can induce certain protective IgM-NAA to become pathogenic.

(i) *Induction of pathogenesis by binding of IgM*-*NAA at cold temperatures*: This is best exemplified in human renal allograft transplant recipients having high IgM-ALA and IgM-anti endothelial cell antibody (IgM-AEA) levels at the time of the kidney transplant. These recipients have a high incidence of delayed kidney graft function (DGF) (161, 162) resulting from high levels of IgM-AEA which cause glomerular endothelial cell injury when, after successful vascular anastomosis, warm blood is allowed to flow into a cold kidney. Fortunately, this self-limiting injury is prevented by warming the kidney prior to re-instituting blood flow. Such observations highlight the nature of IgM-NAA, i.e., their potential for complement mediated cytotoxicity under non-physiological cold conditions (157, 159).
(ii) *Induction of pathogenesis by binding of natural IgM to unmasked neo*-*antigens*. This is best exemplified by the ubiquitous neo-antigen “non-muscle myosin heavy chain type IIA and C (NMM)” which is unmasked in murine models of acute ischemia to the small bowel, skeletal muscle (hind limb) and heart. About 1–2% of IgM-NAA B1 cell clones in mice secrete IgM anti-NMM probably to protect against NMM derived from infectious organisms (163, 164). In murine models of acute ischemia to the bowel or hind limb, injury is predominantly mediated during reperfusion by innate inflammation triggered by IgM binding to the unmasked NMM and activation of complement (6, 163, 165, 166). RAG-1 ko mice are normally protected from this ischemic injury to the small bowel or hind limb (163, 166). However, RAG-1 ko mice succumb to this ischemic injury after infusion of polyclonal IgM or monoclonal IgM anti-NMM. Additionally, one can clearly demonstrate binding of IgM and complement to NMM in bowel epithelial cells or to hind limb striated muscle cells (163, 165).
  Interestingly, one does not observe innate inflammation mediated by IgM anti-NMM after murine renal ischemia even though endothelial cells in glomeruli and peri-tubular capillaries express NMM (167, 168). One possibility is that NMM in the peri-tubular capillaries or in the tubules is not unmasked after ischemia. After renal IRI, most of the ischemia induced kidney injury occurs in the outer medullary renal tubules. However, after renal IRI, one can detect increased IgM binding to glomeruli but not to the extensive network of NMM containing capillaries that surround the tubules or the renal tubules (168). Additionally, depleting B1 cells, decreased binding of IgM to glomeruli, and decreased glomerular injury, but did not protect tubules from inflammation-mediated renal injury, thus indicating that the innate inflammation seen after renal IRI is not mediated by natural IgM and complement (168). Other studies would indicate that the inflammatory response after renal IRI is mediated by ischemia-induced renal tubular injury that activates innate immune cellular mechanisms involving NK and NKT cells (169). This latter study would also explain why Rag-1 ko mice and IgM ko mice without secretory IgM are not protected from renal IRI (4, 138, 170–172).
(iii) *Induction of pathogenesis by non-physiologic expansion of specific IgM*-*NAA clones*. This is best exemplified by expansion of certain B1 cell clones that specifically secrete rheumatoid factor (RhF), an IgM-NAA that binds to self IgG. Excess production of RhF predisposes to generation of large circulating IgM/IgG complexes (referred to as cryoglobulins as these complexes precipitate *ex vivo* in the cold) that cause thrombosis of small blood vessels especially in the kidney glomeruli and skin (173, 174). This serious clinical problem is treated by plasmapheresis (to remove cryoglobulins) and agents to deplete B cells. The most common etiology for monoclonal or polyclonal expansion of RhF secreting B1 cell clones is chronic hepatitis C infection. Currently, it is unclear why this viral infection is associated with clonal expansion of RhF secreting B1 clones. More importantly, even though RhF was the first IgM-NAA to be discovered, we do not understand their normal physiological role.

### Nature of IgM-ALA and Understanding Their Anti-Inflammatory Function

In this section, we review some of our pertinent observations on IgM-ALA that initially were discovered because of their lymphocytotoxic activity at room temperature in the presence of complement [reviewed in Ref. (39)]. Here, we show (a) that a substantial subset (8–10%) of IgM secreting human B cell clones, obtained from the umbilical cord, has IgM-ALA activity, (b) that polyclonal IgM-ALA clones have binding specificity to certain leukocyte subsets and that polyclonal IgM-ALA will bind to only some leukocyte receptors and not others. More importantly, we show that the repertoires of IgM-ALA vary in disease states among different individuals, and (c) that IgM-ALA exhibits anti-inflammatory effects, both *in vitro* and *in vivo*. We show that the anti-inflammatory effects are in part explained by IgM-ALA binding to co-stimulatory receptors and regulating the function of T effector cells and DC without decreasing Tregs. These *in vitro* studies involve human and murine cells, while *in vivo* studies were performed in mice.

#### Human Umbilical Cord B Cell Clones Produce IgM-ALA that Exhibit Leukocyte Receptor Specificity – Binding of IgM to Leukocytes was Not Mediated by FcμR

**Figure 4:**
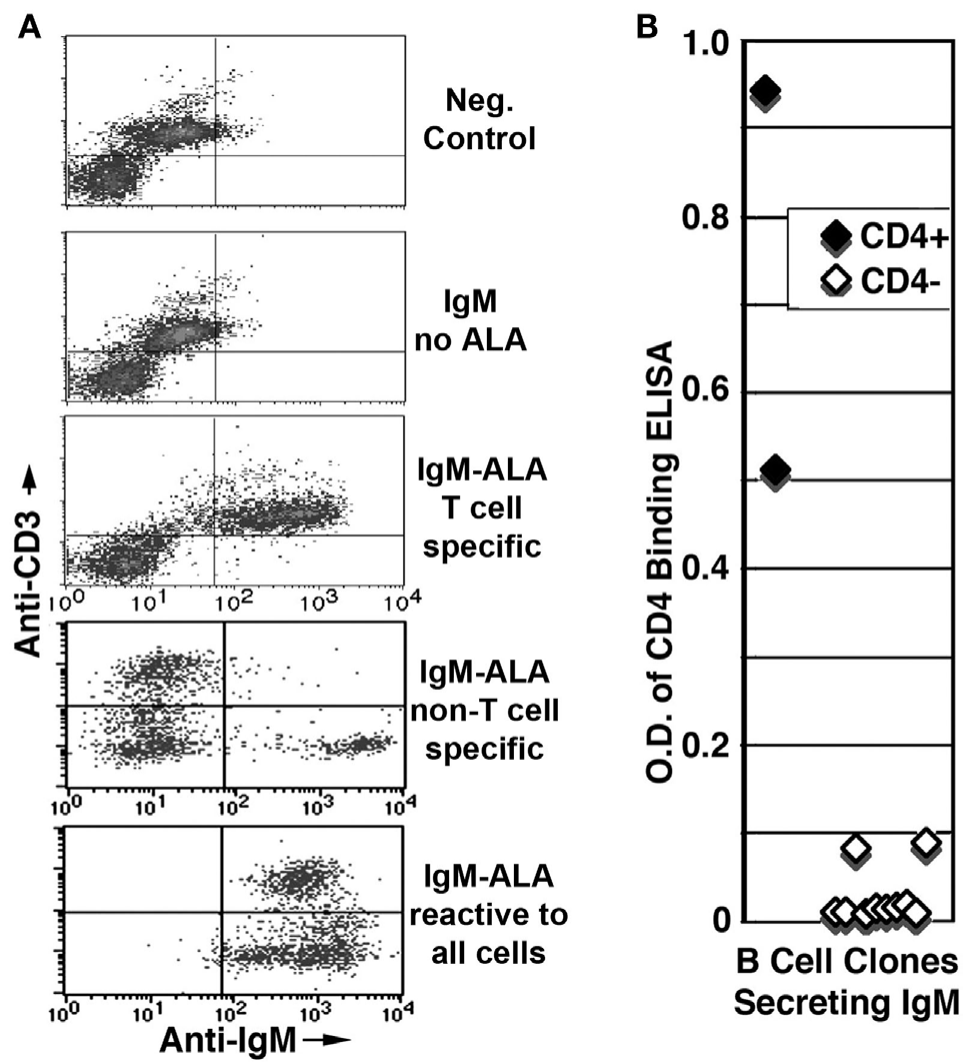
**Presence of IgM-ALA in supernatants from umbilical cord B cells**. **(A,B)** *IgM*-ALA reactivity in IgM containing supernatants from B cell clones activated with the EBV virus. IgM-ALA reactivity was detectable in 8 of 79 supernatants. **(A)** Supernatants were interacted with cells containing a mixture of cell lines, i.e., Jurkat, SupT-1, Daudi, and U937. Note that the supernatants have IgM-ALA that is specific for either T or non-T cells or all four cell lines. Subsequent studies revealed that IgM anti-non-T cell had binding reactivity to only U937 cells and not to Daudi cells. **(B)** Data on IgM anti-CD4 reactivity using an ELISA technique. Note that only 2 of 79 IgM-containing supernatants had IgM anti-CD4 reactivity and both these supernatants also had IgM with binding to leukocytes. Figure and legend reproduced with permission from Ref. (39). Copyright 2008. The American Association of Immunologists, Inc.

We initially wanted to determine if IgM-ALA exhibited leukocyte receptor specificity as natural antibodies are polyreactive and, hence, each monoclonal IgM could non-specifically bind to carbohydrate or other moieties on several leukocyte receptors. We isolated B cell clones from human umbilical cord blood and observed that >90% of B cells were IgM secreting with about 10% of the IgM clones having IgM-ALA binding activity when examined by flowcytometry on a cell mixture comprising of B (Daudi), T (Jurkat, Sup T1), and macrophage (U937) human cell lines. The lack of demonstrable leukocyte reactivity by the majority of IgM clones would indicate that IgM did not bind to FcμR on human B cells and macrophages (175). Second, we observed that these IgM-ALA clones exhibited leukocyte subset specificity in that some of the IgM-ALA monoclonal antibodies only bound to receptors expressed by all leukocytes or either T cells (SupT-1, Jurkat) or macrophages (U937) or B cells (Daudi) (Figure 4) (39). Further studies revealed that only human monoclonal IgM, having T cell reactivity, immunoprecipitated CD4 from cell lysates and bound to recombinant soluble CD4, thus indicating that IgM-ALA can exhibit both receptor and cell specificity.

#### Polyclonal IgM from Different Human Sera Differ in Their Repertoire for Receptor Binding. IgM, in Addition, Regulates Human T Effector Cells and DC Without Affecting Tregs or Chemokine Production

**Figure 5:**
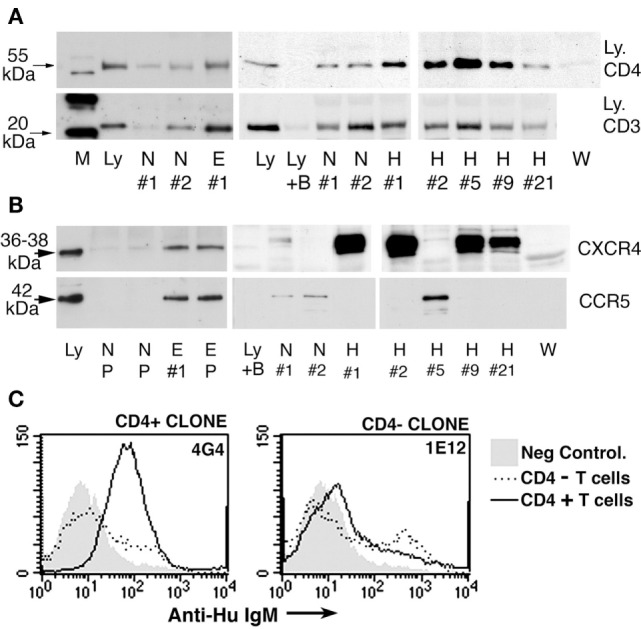
**Immunoprecipitation experiments to show binding of human polyclonal IgM to CD3, CD4, CCR5, and CXCR4**. **(A,B)** Identical quantities of individual (labeled no. 1, 2, etc.) or pooled (labeled P) IgM from normal (labeled N), HIV (labeled H), ESRD (labeled E), or Waldenstrom (labeled W) were used to immunoprecipitate leukocyte receptors from equal amounts of whole cell lysates or recombinant soluble CD4. As controls, Western blots were performed with cell lysates in the absence of agarose beads [to control for binding of primary Ab to leukocyte receptor and to determine receptor size (labeled Ly)]. In another control, agarose beads without IgM were added to lysate to determine whether the leukocyte receptor non-specifically bound to the bead (B plus Ly). Note that several fold more receptors were immunoprecipitated by ESRD and HIV IgM when compared with normal IgM. **(C)** Significantly increased binding of IgM from B cell clone 4G4 to CD4^+^ T cells when compared with CD4^−^ T cells (MCF 71.6 vs. 16.6). Clone 4G4 secreted IgM with anti-CD4 reactivity. Note that no increased binding was observed on CD4^+^ T cells using a B cell clone (IE12) secreting IgM without anti-CD4 reactivity (MCF 22.5 vs. 22.3). Figure and legend reproduced with permission from Ref. (39). Copyright 2008. The American Association of Immunologists, Inc.

We used polyclonal IgM, purified by size exclusion chromatography, from sera of normals, HIV patients and renal dialysis (ESRD) patients (39). Ammonium chloride precipitation was not used as this method affected IgM-ALA binding [see method details in Ref. (39)]. We showed that IgM could immunoprecipitate CD3, CD4, CCR5, and CXCR4 from lysates of cell lines (Figure 5). However, the repertoire of IgM-ALA was found to be different among individuals, especially patients as exemplified with HIV patients (see Figure 5). Such differences in the repertoires of IgM-ALA may explain why clinical manifestations of inflammation are different among different individuals. It is also possible that prior exposure to different infective agents or foreign antigens may explain the observed differences in the repertoire of IgM-ALA among different individuals (31).

**Figure 6:**
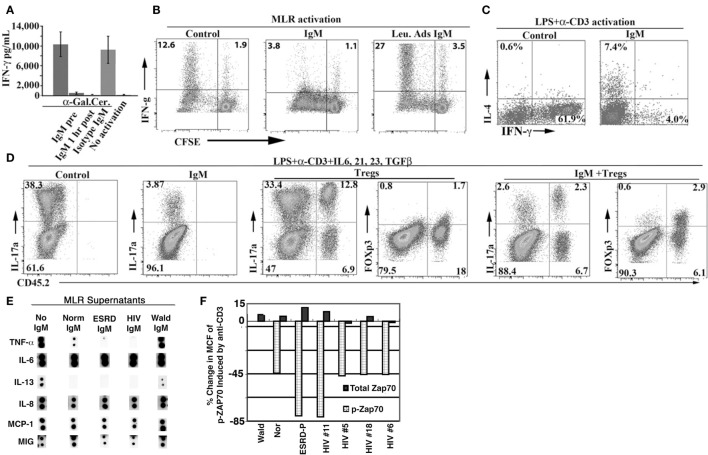
**Polyclonal IgM inhibits IFN-γ production and T cell proliferation and differentiation into TH-1 and TH-17 cells of murine splenic cells and specific pro-inflammatory cytokines from human leukocytes activated with alloantigens**. **(A)** Supernatant IFN-γ in 48 h culture media of splenic cells activated with a-gal-ceramide that specifically activates NKT-1 cells. IgM was added either 0.5 h before activation (IgM pre) or 1 h post activation. **(B–D)** CFSE labeled WT-B6 splenocytes (2.5 × 10^5^ in 0.5 ml media) were activated either in a one-way MLR (using 7.5 × 0^5^ BALB/c irradiated splenocytes) or LPS (350 ng) and soluble anti-CD3. Cells were cultured for 4 to 5 days. IgM (10–15 μg) was added at the initiation of culture unless otherwise indicated. In **(D)**, the effect of Tregs was evaluated by co-culturing 2.5 × 10^5^ CD45.1 WT-B6 splenic leukocytes, containing 1.8% CD4^+^ Foxp3^+^ cells, with 0.5 × 10^5^ CD45.2 WT-B6 Tregs (76% Foxp3^+^) under cytokine conditions favoring TH-17 differentiation. **(E**) Pooled human normal, ESRD, and HIV IgM but not Waldenstrom IgM significantly inhibit the increase in TNF-α and IL-13 but not that of IL-6, IL-8, MIG, and MCP-1 produced in response to alloantigen activation of T cells. Supernatants were obtained from day 5 MLR cultures stimulated in the presence or absence of pooled IgM (15 μg/ml), added on day 0. **(F)** Pooled human IgM inhibits anti-CD3-mediated Zap-70 phosphorylation of human T cells. IgM was added 30 min before anti-CD3/28 and cells were cultured overnight before quantitation with flowcytometry. Figures and legend reproduced with permission from Ref. (138) **(A–D)** and (39) **(E,F)**. Copyright 2008 and 2012. The American Association of Immunologists, Inc.

In *in vitro* studies with human peripheral blood mononuclear cells (PBMC), addition of polyclonal human IgM, but not human IgG or Waldenstrom’s IgM lacking IgM-ALA, differentially inhibited co-stimulatory receptor upregulation, cytokine production, and proliferation of T cells (39). Both normal and patient IgM downregulated expression of CD4, CD2, and CD86 but not CD8 and CD28 on blood PBMC activated with alloantigens (MLR) (39). Additionally, both normal and patient IgM inhibited production of the same set of cytokines, i.e., TNF-α, IL-13, and IL-2 but not IL-6 and chemokines when human PBMC were activated by alloantigens [Figure 6E; Ref. (39)]. Other investigators working with a monoclonal IgM-ALA with reactivity to TcR have shown that IgM-ALA can inhibit IL-2 production and T cell proliferation by binding to the TcR (5, 176). Similarly, we showed that IgM inhibited T cell proliferation induced by alloantigens or CD3 ligation and also inhibited Zap-70 phosphorylation induced by anti-CD3 [Figure 6F; Ref. (39)]. However, IgM did not alter chemokine production by activated PBMC but inhibited chemokine-induced chemotaxis by binding to the receptor and blocking chemokine binding (39). Importantly, IgM did not alter Treg levels in an MLR, despite inhibiting T cell proliferation. In general, identical quantities of patient IgM had a more inhibitory effect in the above studies when compared to normal IgM (39). These functional differences between normal and patient IgM may be explained by differences in their quantity and IgM-NAA repertoire (Figure 5). Other investigators have also shown that polyclonal human IgM can inhibit proliferation of human T cells (5, 177).

In summary, the data indicate that polyclonal IgM, in physiological doses, inhibit human T effector cell activation and proliferation as well as regulates production of certain cytokines by binding to certain co-stimulatory molecules (CD4, CD3, TcR). IgM does not inhibit T regs. We also show that the quantity and repertoire of IgM-ALA varies in different individuals especially in disease. Interestingly, IgM-ALA does not appear to affect the production of chemokines by leukocytes, but interferes with their action by binding to chemokine receptors.

#### Polyclonal Murine IgM Binds to Specific Co-Stimulatory Receptors and Regulates the Function of Murine T Effector Cells, DC, and NKT Cells but Not Tregs

**Figure 7:**
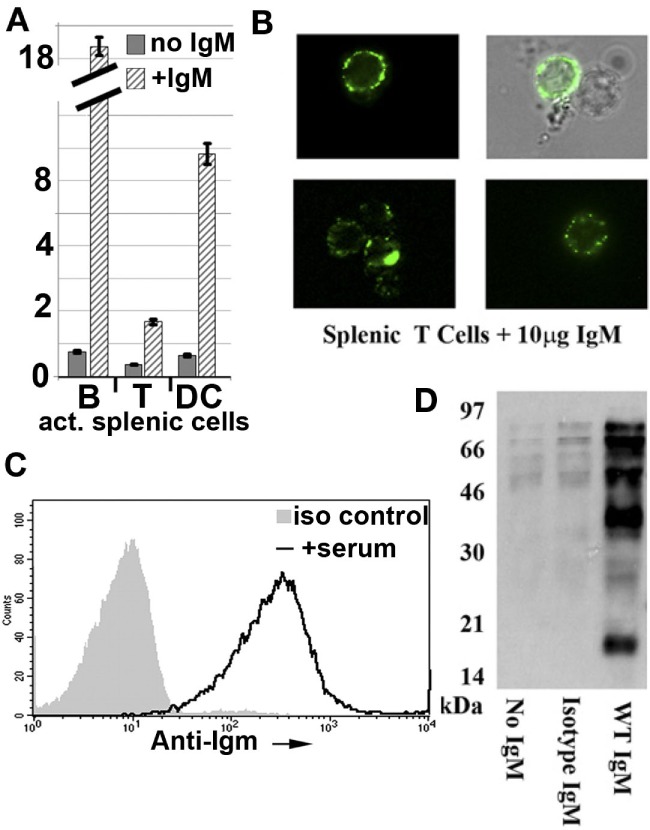
**Polyclonal murine WT IgM bind to membrane receptors on leukocytes**. **(A)** Polyclonal IgM has several fold increased binding to activated murine splenic B cells and dendritic cells (DC). Splenic leukocytes, activated for 48 h were interacted with purified mouse IgM at 4°C and evaluated for IgM binding using IgG anti-IgM (clone 11/41). Isotype monoclonal IgM with reactivity to KLH did not bind to activated leukocytes (data not shown). IgM binding to B cells was evaluated by blocking intrinsically expressed IgM with unlabeled IgG anti-IgM (clone 11/41). **(B)** It depicts immunofluorescence microscopy images of IgM binding to cell membranes of splenic T lymphocytes. **(C)** It compares binding of IgM and isotype IgM on CD3^+^WT-B6 pronase pretreated splenic leukocytes. Spleen cells were pronase digested to remove FcμR and to show that IgM-ALA can bind to other receptors on cell membranes. **(D)** It depicts a representative example of a Western blot from two separate experiments demonstrating immunoprecipitation by WT-polyclonal IgM of biotinylated membrane proteins from the murine macrophage cell line J77. In this experiment, WT-polyclonal IgM is compared with an equal amount of isotype IgM that has no binding activity to leukocytes using flow cytometry. Figure and legend reproduced with permission from Ref. (4) **(A)** and Ref. (138) **(B–D)**. Copyright 2012 and 2015. The American Association of Immunologists, Inc.

In murine studies, we initially observed that IgM-ALA bound to pronased splenic leukocytes (Figures 7B,C) and that IgM-ALA had several fold increased binding to live splenic granulocytes, DC, and B cells when compared to T cells and IgM binding to all leukocytes was enhanced when cells were activated (see Figure 7A). Furthermore, we showed that IgM-ALA bound to splenic leukocytes independently of FcμR and, second, showed that IgM, after binding to leukocytes, could immunoprecipitate several different leukocyte receptors (Figure 7D) (138). These observations, together with the above findings with human leukocytes, led us to investigate if IgM had an inhibitory effect on the function of T cells, DC, and NKT cells by binding to receptors, e.g., antigen-presenting receptors and co-stimulatory receptors, that get upregulated during activation. Both T cells and DC have an important role in adaptive immunity, e.g., in transplantation and NKT cells, together with DC, have an important role in innate immunity.

With murine leukocytes, we showed in *in vitro* studies, that physiological doses of murine polyclonal IgM inhibited naïve T cells from differentiating into TH-1 and Th-17 cells even when IgM was added 48 h after activation (Figures 6B–D) (138). This inhibitory effect of IgM-ALA on T cells did not depend on presence of DC as the same inhibitory effect was noted when T cells were activated with insoluble anti-CD3/28. IgM inhibition of anti-CD3-mediated Zap-70 phosphorylation would indicate that the inhibitory effect of IgM on T cell function is mediated by IgM binding to CD3 [Figure 6F; Ref. (39)]. IgM did not, however, inhibit differentiation of murine T cells into Foxp3+ cells and, furthermore, under Th-17 differentiating cytokine conditions, IgM inhibited sorted Foxp3+ cells from differentiating into TH-17 cells (Figure 6D) (138).

**Figure 8:**
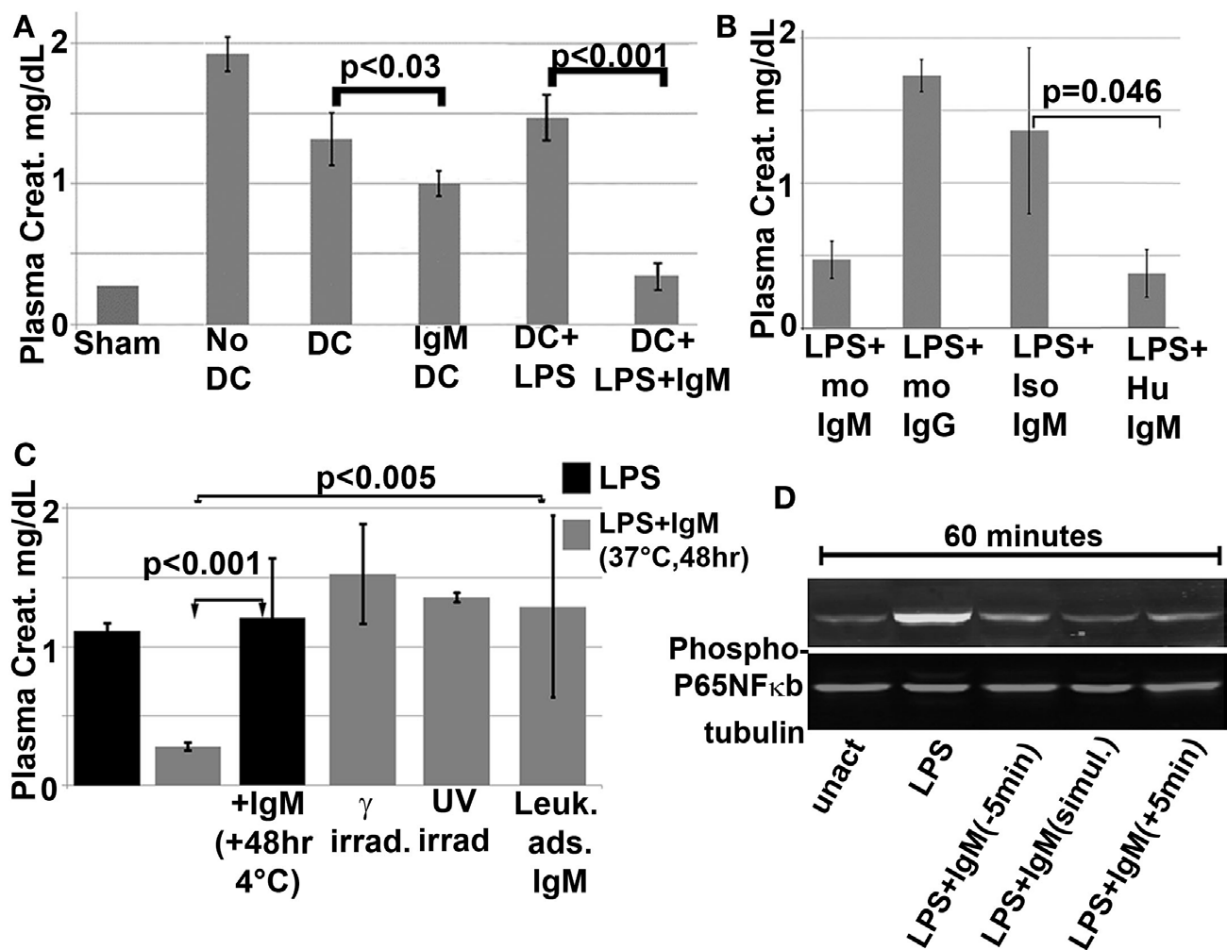
***In vivo* infusion of polyclonal human or murine IgM pretreated WT-BMDC with downregulated NF-κB protects mice from renal IRI**. **(A)** There is more protection when using WT-BMDC pretreated with both IgM and LPS as evaluated by SCr. WT-BMDC were pretreated with IgM for 1 h before adding LPS and culturing BMDC at 37°C for 48 h. **(B)** LPS-activated BMDC pretreated with either mouse or human IgM are protective in renal IRI. **(C)** Pretreating 48 h LPS-activated WT-BMDC with IgM for 1 h at 4°C is not protective in IRI. Fourty-eight hour IgM/LPS-pretreated WT-BMDC are non-protective in IRI after being irradiated (3000 Rad) or rendered apoptotic by exposure to UV light. Additionally, polyclonal IgM adsorbed with splenic leukocytes to deplete IgM-ALA clones was not protective in IRI. **(D)** Western blot depicting IgM-mediated downregulation of LPS-induced phosphorylation of p65 NF-κB in BMDC. IgM was added either before or after LPS activation. Figure and legend reproduced with permission from Ref. (4) Copyright 2015. The American Association of Immunologists, Inc.

Since there are <1.5% of DC in murine splenic leukocytes, we used 7–8 day cultured murine bone-marrow DC (BMDC) to investigate the functional effects of IgM on DC (4). We showed that polyclonal murine IgM, but not IgM pre-adsorbed with activated splenic leukocytes, bound to recombinant soluble CD40 and PD1 but not PDL-1, CD40L, and CD80, indicating, therefore, that IgM-ALA has binding specificity to certain DC receptors, just as we observed with human T cell receptors where IgM bound to CD4, CD3, and CD2 but not to CD8 (39). The functional effect of IgM binding to certain specific co-stimulatory receptors was tested on LPS-activated BMDC. We show that IgM, even when added 2 h after LPS, inhibited LPS-induced CD40 upregulation, but not upregulation of CD86, PDL-1, and MHC-II of BMDC and downregulated basal expression of PD1 on BMDC. IgM, in addition, downregulated p65NF-κB activation induced by LPS (Figure 8D) but not by LPS + anti-CD40 (agonistic Ab), thus indicating that IgM can inhibit p65NF-κB upregulation mediated by TLR4 activation, but not when both TLR4 and CD40 are activated (4). Interestingly, IgM inhibited TLR4 activation by a mechanism that did not involve inhibition of LPS binding to cell receptors (4). However, despite downregulation by IgM of TLR4-induced p65NF-κB, there was no decrease in IL-12 production or increase in IL-10 production indicating that LPS activates other transcription factors, besides p65NF-κB, to upregulate these cytokines and certain other co-stimulatory receptors that were not downregulated with IgM (4). In *in vivo* studies (to be presented in the next section), we show that IgM pretreatment of LPS-activated BMDC switches these activated BMDC to a regulatory phenotype possibly by a mechanism involving downregulation of CD40 and NF-κB.

We next did *in vitro* studies to test the effect of polyclonal IgM on Type1 NKT cells, which together with DC, have an important role in inducing innate inflammation, e.g., after renal reperfusion injury (IRI). In these studies, we used α-gal-ceramide, a glycolipid that is taken up by DC and presented via the CD1d receptor to Type 1 NKT cells, which get activated and secrete IFN-γ. This assay is specific for determining Type 1 NKT function as only Type 1 NKT, but not T effector cells, will secrete IFN-γ after exposure to α-gal-ceramide, which is recognized, in context of CD1d presentation, by the invariant TcR on Type 1 NKT cells. We show that physiological doses of IgM inhibits α-gal-ceramide induced IFN-γ production of splenic leukocytes even when IgM is introduced 1 h after α-gal-ceramide (Figure 6A) (138). We have not defined the mechanism for the inhibitory effect of IgM on Type 1 NKT function.

In summary (see Table 2), the *in vitro* data with both human and murine cells would indicate that IgM-ALA regulates leukocyte function by binding and downregulating certain leukocyte receptors (e.g., CD4 and CD2 on T cells, CD40, and CD86 on DC) and inducing regulatory function in DC. Importantly, IgM does not decrease Foxp3+ Tregs. IgM regulates leukocyte activation, proliferation, and chemotaxis to attenuate excess inflammation [Figure 6; Ref. (39)]. The marked individual variation in the repertoire of IgM-ALA, with specificity to the different leukocyte receptors, observed in both normal and disease states could potentially explain the differences in the vigor and character of inflammatory responses in different individuals exposed to the same inciting agent. Additionally, differences in inflammatory response may also be influenced by total levels of IgM-NAA or IgM-ALA as we observed in transplant recipients (Figure 3). Finally, IgM-ALA, by binding to leukocyte receptors and inhibiting cell activation, can provide another mechanism to limit viral entry into cells and replication as we have shown with the HIV-1 virus (Figure 2) (144).

**Table 2:** ***In vitro* effects of IqM-ALA on human and murine leukocytes**.

	T cells	NKT-1 cells	BMDC
Degree of membrane binding	Moderate	Moderate	Very high
Cell receptor binding	CD4, CD3, TcR, downregulation of CD4; inhibits HIV entry	CD4	Binds CD86, CD40
Intracellular signaling	Inhibits ZAP-70 activation		Inhibits LPS-induced NFκB activation
Pro-inflammatory mediators	Inhibits production of IFNγ, IL-17, TNFα, IL-2	Inhibits production of IFNγ	No effect on IL-12 or IL-10
Anti-inflammatory mediators	Enhances production of IL-4, enhances T regs		Switches BMDC to regulatory phenotype (PD-1, IL-10 dependent)
Proliferation	Inhibits proliferation (alloantigen and anti-CD3/28)		

#### IgM-ALA Inhibits the Innate Immune Inflammatory Response in Renal Ischemia Reperfusion Injury

To test the *in vitro* inhibitory effects of IgM-ALA on DC and NKT cells, we used an *in vivo* murine model of renal IRI (169). In this model, renal vessels to both kidneys are completely occluded with clamps for 26 or 32 min to induce either mild or severe ischemic renal tubular injury. The kidneys are then allowed to re-perfuse by unclamping the blood vessels and the extent of renal injury or decrease in renal function is quantitated at 24 h of reperfusion by measuring for accumulation in the plasma of waste products (e.g., creatinine) that are normally only removed by the kidneys. In this model, the initial ischemic injury is not sufficient to impair renal function as quantitated by measuring plasma creatinine, but it is the innate inflammatory response to products released by ischemic renal cells (e.g., DAMPS and glycolipids) that significantly worsen kidney injury that leads to loss of function. In this model, DAMPS and glycolipids released by ischemic renal cells are taken up by DC and in the splenic marginal zone, DC present glycolipids in the context of CD1d to activate NKT cells, which rapidly release IFN-γ to activate innate effector cells especially granulocytes, macrophages, and NK cells (169). Activated innate effectors migrate to the kidney, where chemokines, released by ischemic cells, enhance extravasation of inflammatory cells into the kidney interstitium. The inflammatory effector cells in the kidney interstitium cause further renal tubular injury with loss of kidney function that leads to an increase in plasma creatinine. This acute loss in kidney function is referred to as acute kidney injury (AKI).

**Figure 9:**
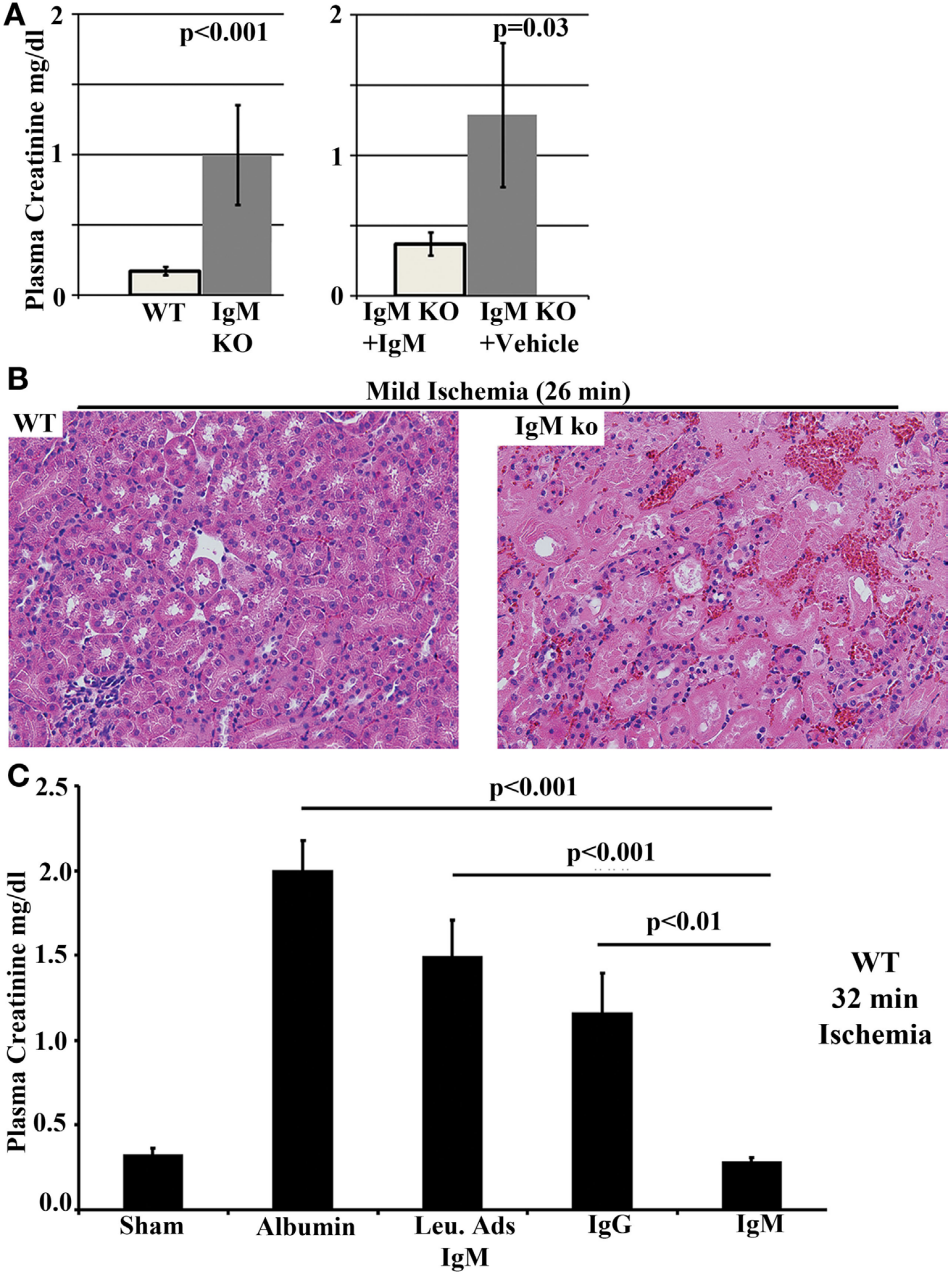
**B6/S4-IgMko mice are more sensitive to renal IRI when compared to their WT counterparts (WT-B6/S4)**. **(A, B)** Kidneys from B6/S4-IgMko mice and their WT counterparts (WT-B6/S4) were subjected to mild ischemia (26 min) and then reperfused. Data depict 24 h plasma creatinine comparing (WT-B6/S4) WT mice with B6/S4-IgMko mice, and B6/S4-IgMko pretreated with 240 μg IgM, 24 h before ischemic injury. Histology depicts H&E staining of renal outer medulla after 24 h of reperfusion. **(C)** Polyclonal IgM, but not leukcocyte adsorbed IgM (Leu-Ads IgM), protects against renal IRI in WT-B6 mice. In these studies, WT-B6 mice were pretreated with equal quantities (150 μg in 0.75 ml) of IgM or Leu-Ads IgM or IgG, 24 h before subjecting the kidneys to severe ischemia (32 min). Kidneys were reperfused for 24 h prior to determining plasma creatinine **(C)**. Control mice were pretreated with 0.75 ml RPMI containing 150 μg bovine albumin to exclude variables, such as volume/colloid, that can protect against ischemic injury. Figure and legend reproduced with permission from Ref. (138). Copyright 2012. The American Association of Immunologists, Inc.

We used two approaches to test the protective role of IgM in the suppression of this ischemia-induced innate inflammatory response. First, we performed renal IRI in B6/S4-IgMko mice (referred to as IgM ko) that lack circulating IgM but have normal levels of other immunoglobulins. These mice have normal or increased levels of Tregs, B regs, and IL-10 and their normal functioning B cells express membrane IgM or BcR but are unable to secrete IgM. Unlike their WT counterpart, we demonstrated that these mice are very sensitive to renal ischemia, developing AKI with mild ischemia (26 min clamp time) that is insufficient to cause AKI in their WT counterparts (Figures 9A,B). Replenishing IgM in the IgM ko mice with a single 240 μg dose of polyclonal IgM, to achieve plasma levels similar to that in their WT counterparts, protected these IgM ko mice from developing AKI with mild ischemia, thus indicating that sensitivity to ischemia in the IgM ko mice resulted from a lack of circulating IgM (Figures 9A,B).

In the second approach, a single dose (150 μg) of normal purified polyclonal IgM was administered intravenously to wild-type C57BL6 (WT-B6) mice to increase baseline circulating IgM by about 30–50% and to determine if increasing IgM would protect these WT-B6 from severe renal ischemia (32 min clamp time). These studies clearly indicated that increasing circulating IgM levels protected mice from severe renal IRI (Figure 9C). We next determined that this protection was mediated by IgM-ALA as administering similar quantity of polyclonal IgM pre-adsorbed with activated splenic leukocytes to remove IgM-ALA failed to protect these WT-B6 mice from severe renal IRI (Figure 9C).

In both approaches, physiological doses of intravenous polyclonal IgM mediated protection by decreasing the innate ischemia-induced inflammatory response. Protected kidneys had a very minimal inflammatory response with no or minimal tubular injury that could be detected on histology. Based on our *in vitro* data, *in vivo* IgM-ALA could mediate protection through several mechanisms, including regulation of NKT and DC and maintaining or enhancing Tregs, which also mediates protection in this model of innate inflammation (178). Our prior observations showing that IgM-ALA had several fold increased binding to splenic DC, when compared to T cells, prompted us to investigate the role of IgM-ALA in regulating DC in this model. These studies are presented in the next section.

#### Protection from Renal Ischemia is Mediated by IgM Induced Regulatory DC. Regulatory DC Require Tregs, B cells, Circulating IgM, and IL-10 to Mediate *In Vivo* Protection

**Figure 10:**
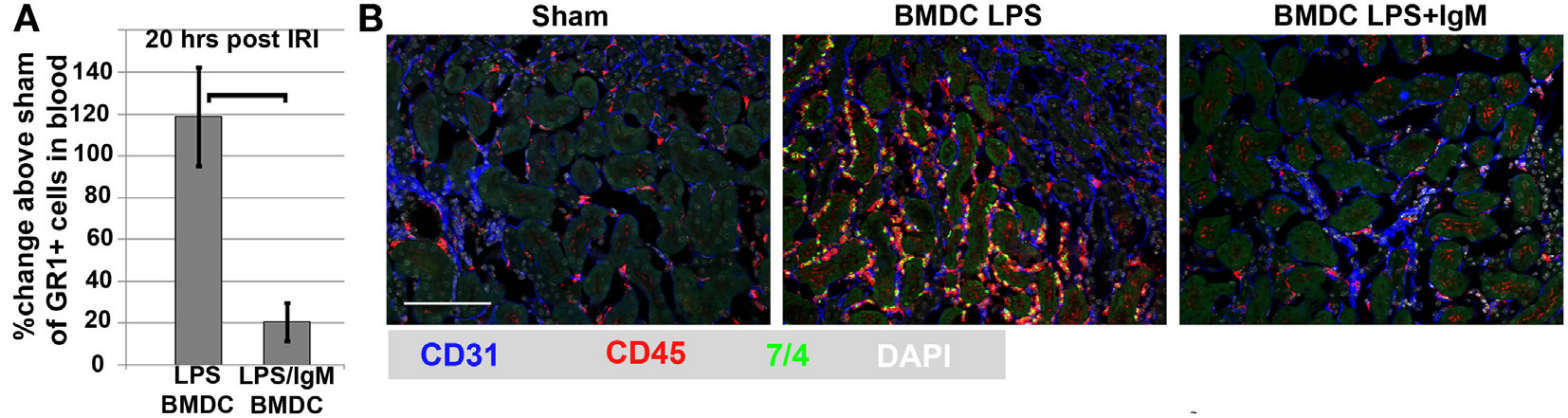
**Murine IgM/LPS-pretreated BMDC inhibit the inflammatory response after renal IRI**. There is significantly less inflammatory cell response 20 h post ischemia in mice administered IgM/LPS-pretreated BMDC as determined by circulating granulocytes (CD45+, GR1+) in blood **(A)** and infiltrating CD45+ and GR1+ leukocytes in kidneys **(B)**. CD31 is a marker for endothelial cells. 7/4 is a marker for GR1+ granulocytes. Figure and legend reproduced with permission from Ref. (4). Copyright 2015. The American Association of Immunologists, Inc.

To examine the *in vivo* role of IgM on DC, we used 7 to 8-day-old cultured BMDC. In these studies, BMDC were activated *ex vivo* for 48 h with LPS with or without polyclonal IgM. Activated BMDC were washed and then 0.5 × 10^6^ BMDC were infused intravenously into mice 24 h before performing renal ischemia. In these studies, IgM + LPS-pretreated BMDC protected mice from ischemia induced AKI (Figures 8A) by inhibiting the increased generation of circulating granulocytes and inhibiting innate activated leukocytes from infiltrating the ischemic kidney [Figure 10, Ref. (4)]. Importantly, protection with LPS-activated BMDC was only observed when IgM was present during the 48-h culture and not when IgM was added at the end of the 48 h LPS activation (Figure 8C), indicating therefore that regulation of BMDC by IgM is an active process requiring both NF-κB and CD40 downregulation induced by IgM (Figure 8D). Preventing downregulation of NF-κB and CD40 by adding the agonistic anti-CD40 antibody to LPS + IgM during activation, negated the protective effect, thus supporting that NF-κB and CD40 downregulation are required to switch activated BMDC to a regulatory phenotype (4). It is possible that IgM by binding to CD40 induces this regulatory phenotype. Ex-vivo pretreatment of murine BMDC with human IgM was also protective in renal IRI (Figure 8B), indicating that the function of IgM-NAA is evolutionarily conserved among species.

However, since LPS activation of BMDC generates apoptotic cells, it is possible that protection from ischemia could be mediated by complexes of IgM anti-PC and apoptotic cells, which when injected, regulate endogenous DC (127). Such a possibility seemed unlikely, as in the *in vivo* murine studies to demonstrate the anti-inflammatory role of apoptotic cells, large quantities of apoptotic cells (2.5 × 10^7^ thymocytes) were used (127), while in our studies, we only used 0.5 × 10^6^ BMDC (4). However, to exclude the possibility of apoptotic cell-mediated protection, we used apoptotic BMDC by subjecting activated LPS + IgM pretreated BMDC (0.5 × 10^6^ cells) to UV irradiation. Such apoptotic LPS + IgM pretreated BMDC failed to protect mice from ischemia induced AKI, thus excluding the role of apoptotic cell/IgM complexes in inducing protection (Figure 8C). The latter experiments indicated that IgM-ALA mediated protection by switching LPS-activated BMDC to a regulatory phenotype. BMDC required IL-10 but not IDO (indoleamine 2, 3-dioxygenase) to switch to a regulatory phenotype as IgM + LPS pretreatment of IL-10 ko BMDC, but not IDO ko BMDC, failed to protect mice from developing AKI after renal ischemia (4).

We hypothesize that intravenously injected regulatory BMDC inhibit the innate inflammatory response by entering the splenic marginal zone where they inhibit NKT function. However, in further studies, we show that injected regulatory BMDC require the presence of other *in vivo* suppressive mechanisms, such as circulating IgM, IL-10, Tregs, and B cells, to mediate protection (4).

In summary, IgM-ALA inhibits the ischemia-induced innate inflammatory response by several mechanisms, including switching activated DC to a regulatory phenotype, inhibiting NKT cell IFN-γ production, and inhibiting chemotaxis of leukocytes by binding to chemokine receptors. However, IgM-ALA is in-effective on its own in inhibiting the ischemia-induced innate inflammatory response and requires the presence of other *in vivo* suppressive mechanisms, such as IL-10, Tregs and B cells. Conversely, these other *in vivo* suppressive mechanisms, such as Tregs and Bregs, cannot effectively protect against ischemia induced innate inflammation without IgM-NAA as evidenced in our renal ischemia experiments using IgM ko mice that lack secretory IgM (Figures 9A,B).

#### Polyclonal IgM Inhibits Inflammation Mediated by Adaptive Immune Mechanisms in Allograft Transplantation

**Figure 11:**
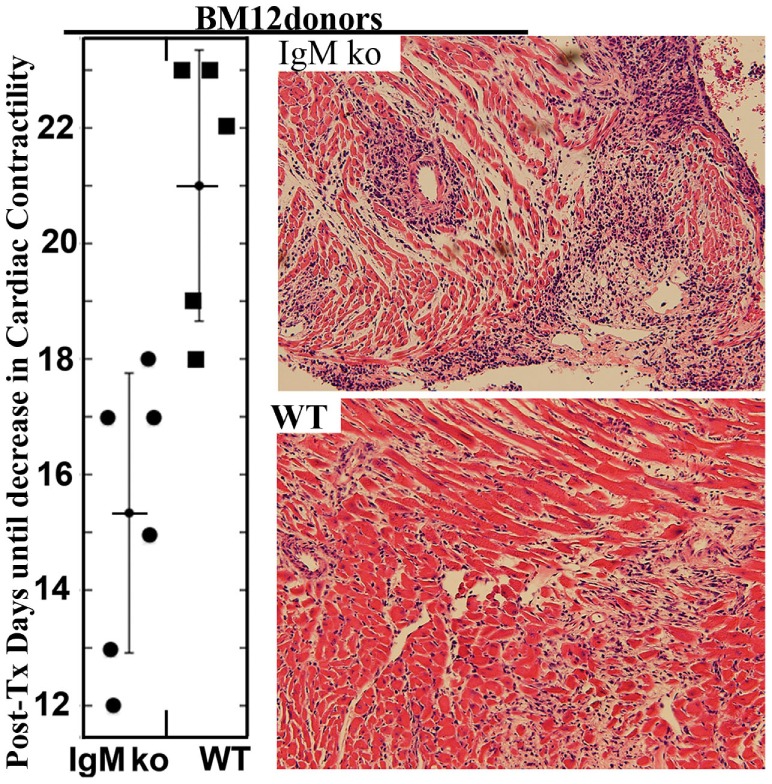
**Allograft rejection is more rapid and severe in B6/S4-IgMko mice**. B6/S4-IgMko mice and their WT counterparts (WT-B6/S4) received cardiac allografts from B6-bm-12 donors that are only incompatible at the MHC-Ia locus. Graph depicts the post-transplant day when cardiac contractility was found to be decreased by finger palpation. Histology depicts Day 10 post-transplant B6-bm12 cardiac allograft histology. Figure and legend reproduced with permission from Ref. (138). Copyright 2012. The American Association of Immunologists, Inc.

Because of our clinical observations (Figure 3) and the *in vitro* studies demonstrating that IgM (a) inhibited alloantigen-activated T cell proliferation and differentiation into Th-1 and Th-17 independently of DC (Figure 6) and (b) could induce regulatory function in DC, we performed experiments aimed at determining whether IgM could also inhibit allograft rejection, which is an *in vivo* model of inflammation mediated by alloantigen-activated DC and T cells (138). Two approaches were used to test the role of polyclonal IgM. First, cardiac transplants were performed intra-abdominally in B6/S4-IgM ko mice (referred to as IgM ko) or their WT littermates using B6-bm12 donor hearts, which are minimally incompatible at the MHC class II locus (Ia) with the recipient. In this transplant model, there is a mild chronic form of cellular rejection and a vasculopathy that is initiated by a T cell-mediated inflammatory process and cardiac graft loss (defined as loss of intra-abdominal cardiac pulsation) occurs at >2 months in WT recipients. However, in IgM ko recipients, there is a more severe acute cellular rejection and graft loss occurs in 2–3 weeks, which is significantly earlier compared with their WT-B6/S4 counterparts, where cardiac graft loss occurs after >2 months (Figure 11). Histologically, there are considerably more TH-17 cells infiltrating the cardiac allograft in the IgM ko recipient despite no significant difference in infiltrating Tregs between the groups (138). The T cell findings on histology mirror the *in vitro* studies where IgM inhibited naïve T cells and Foxp3+ T cells from differentiating into TH-17 cells without affecting levels of Tregs (Figure 6D).

In the second approach, we wanted to determine whether increasing circulating levels of IgM in WT-B6 mice inhibited the severe and rapid rejection that occurs in the setting of fully MHC-incompatible donor hearts (i.e., from BALB/c donors). In this model, rejection in WT-B6 recipients is detectable by day 5 and graft loss occurs by 7–9 days (138). In these studies, 175 μg IgM was intravenously administered 24 h after ascertaining that cardiac surgery was successful, and the dose of IgM was repeated on days 3 and 5. Mice were euthanized on day 6. The data clearly show that IgM inhibited the severe inflammation in the cardiac allograft induced by rejection on day 6, as detected by H&E staining and immunofluorescence staining for neutrophils (7/4) and T cells (CD3). Importantly, with immunohistochemistry, this lack of leukocyte infiltration in the cardiac parenchyma of IgM-treated recipients was also associated with no or minimal CXCL1+ leukocytes and with no or minimal fragmentation of capillaries, as identified by the endothelial cell marker CD31 (138).

In summary, we show that physiological doses (175 μg) of polyclonal IgM can subdue inflammatory responses mediated by an adaptive immune mechanism. Potential mechanisms include the following (a) a direct inhibitory effect of IgM-ALA on T effector cells, but not Tregs, possibly by binding and down-modulating CD3/TcR and certain specific co-stimulatory receptors, such as CD4 and CD2 but not CD8. As a result, T effector cells are inhibited from proliferating or producing certain specific cytokines (e.g., TNF, IFN-γ, IL-17 but not IL-6, and chemokines) or from differentiating into TH-1 and TH-17 pro-inflammatory cells. Importantly IgM-ALA does not affect levels of Foxp3+ Tregs, but prevents Foxp3 + cells from differentiating into TH-17 cells under pro-inflammatory conditions, (b) by binding to CD40 and switching activated DC to a regulatory phenotype with downregulation of CD40 and p65NF-κB, and (c) by inhibiting chemotaxis. It is highly unlikely that IgM anti-PC could have a significant role in inhibiting allograft rejection in our studies as we used small doses of polyclonal IgM (175 μg) while 1.5–2.0 mg of a monoclonal IgM anti-PC (T15 idiotype) was used to inhibit an arthritis model of inflammation mediated by adaptive immune mechanisms (127).

#### Polyclonal IgM Antibodies Inhibit Autoimmune-Mediated Insulitis in NOD Mice

Insulitis in the NOD mouse is primarily mediated by autoimmune T cells but there are data to indicate that B cells are also involved. Depleting B cells or only B1 cells ameliorates insulitis indicating that some of the IgG autoantibodies detected in NOD mice may also be pathogenic (179–181). The role of B1 cells in generating pathogenic IgG autoantibodies has also been described in other autoimmune murine models (20, 94). Because our *in vitro* studies demonstrated that polyclonal IgM inhibited T cell proliferation and differentiation into Th-1 and Th-17 cells (Figures 6B–D) and can also counter pathogenic IgG autoantibodies (as previously discussed), we performed studies to determine whether IgM could inhibit autoimmune insulitis that results in islet cell destruction and diabetes mellitus (DM) in NOD mice (182). In these mice, the autoimmune inflammatory process begins spontaneously around 4–5 weeks after birth and the initial phase is characterized by a silent and non-destructive leukocyte infiltration of the perivascular and periductal regions in the pancreas as well as the peripheral islet regions by a heterogeneous mixture of CD4 and CD8 T cells, B cells, macrophages, and DC (peri-insulitis). In the invasive phase that begins at 8–12 weeks of age, the immune infiltrate enters the islet inducing beta cell destruction (insulitis). Significant destruction first becomes evident around 12–13 weeks of age with mice exhibiting overt diabetes (DM).

We wanted to determine the effect of increasing IgM levels on development of DM (182). NOD mice were administered bi-weekly intra-peritoneal polyclonal IgM (50 μg/dose) beginning either at 5 or 11 weeks of age and ending when mice were 18 weeks old. At 25 weeks of age, 80% of control mice (*n* = 30) became diabetic, while 0% of mice (*n* = 30) treated with IgM beginning at 5 weeks developed DM. Importantly, only 20% of pre-diabetic mice (*n* = 20) treated with IgM beginning at 11 weeks of age developed DM at 25 weeks of age. This latter observation is particularly notable as prior studies using co-stimulatory blockade failed to prevent DM in this murine model. At 18–25 weeks of age the pancreas revealed no or minimal insulitis in NOD mice treated with IgM beginning at 5 weeks of age. Other investigators using monoclonal polyreactive natural IgM in the neonatal period have also obtained similar results (183, 184).

In summary, these studies indicate that polyclonal IgM inhibits insulitis via several potential mechanisms, including inhibition of autoimmune T effectors and possibly countering IgG autoantibodies via anti-idiotypic mechanisms and by inhibiting the B cells that produce them. Additionally, IgM by switching activated DC to a regulatory phenotype and maintaining Tregs could enhance this protective effect.

## Conclusion

**Figure 12:**
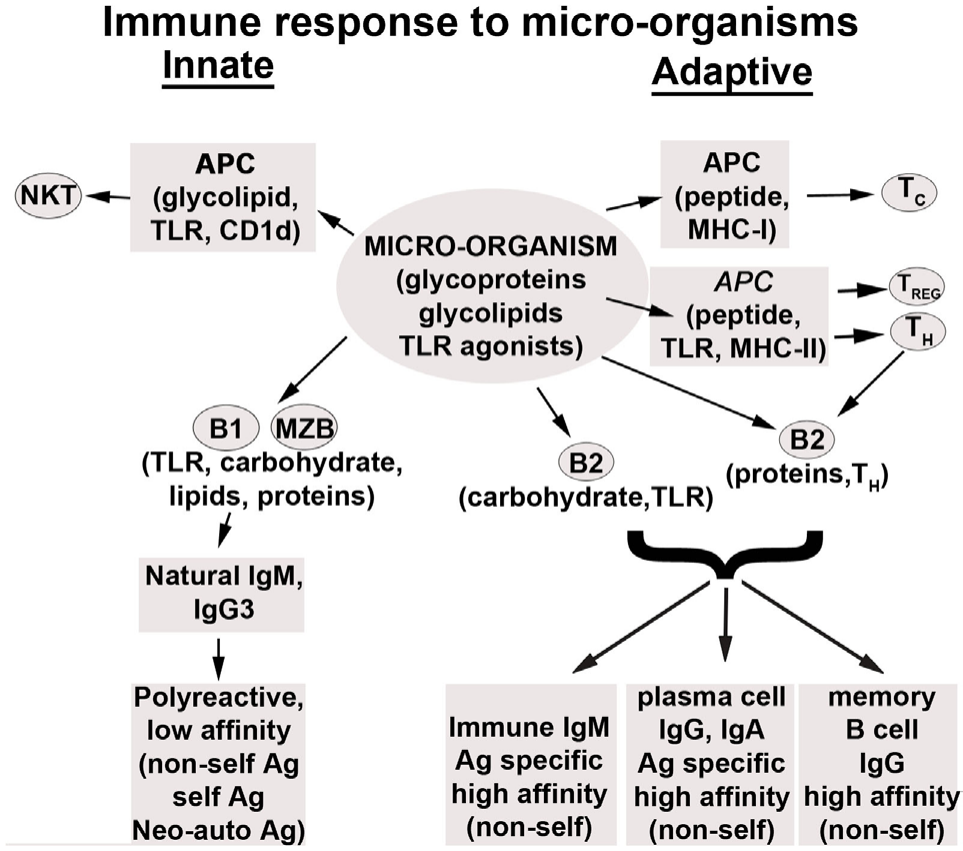
**Immune response to micro-organisms**.

**Figure 13:**
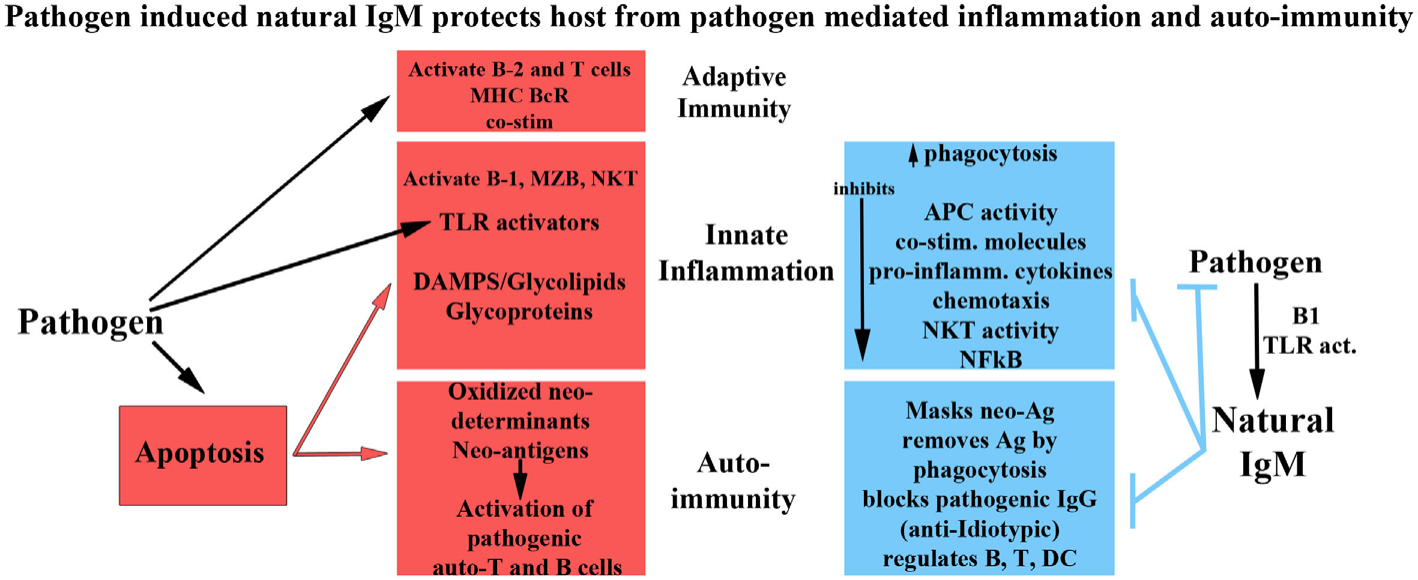
**Pathogen-induced natural IgM protects host from pathogen-mediated inflammation and autoimmunity**.

Figures 12 and 13 summarize our concepts of the inter-relationship between pathogens and natural antibodies. In both murine and human models, the evidence shows that these polyreactive and low-affinity binding IgM-NAA function under physiological conditions to (i) provide a first line of defense against invading organisms, (ii) protect the host from autoimmune inflammation mediated by autoimmune B and T cells that have escaped tolerance mechanisms, (iii) protect the host from endogenous oxidized neo-determinants and other neo-antigens that are unmasked during tissue damage, and (iv) regulate excess inflammation, mediated by both innate and adaptive immune mechanisms. Even though the full repertoire of IgM-NAA develop during the first few years of life, both their levels and repertoire differ in healthy individuals as well as in disease and could contribute to the varying inflammatory response, for example, after an infection or alloantigen exposure (see Figures 2 and 5). We hypothesize that high protective levels of IgM-NAA are maintained by infections that have been shown to increase IgM-NAA, especially IgM-ALA and anti-PC [reviewed in Ref. (31, 39, 148)]. Such a hypothesis could explain the significantly low incidence of autoimmune disorders, such as SLE or sarcoidosis in rural parts of Africa where malaria and other infections are endemic (185–187). We have shown that IgM-ALA increases in active sarcoidosis (188). There are other suppressive mechanisms (e.g., Tregs, B regs, IL-10, TGF-β) that regulate inflammation and based on the different animal models of inflammation, it would appear that IgM-NAA have a more prominent role in regulating inflammation that involves pathogenic IgG autoantibodies, macrophages, and NKT cells. However, we show that IgM-NAA require Tregs, B cells, and IL-10 to be fully effective in controlling inflammation (4). Conversely, our studies and that of others, using mice deficient in IgM secretion, would also indicate that Tregs and Bregs also require IgM-NAA to effectively control inflammation (39, 119, 120). Finally, it may be easier to develop a vaccine to increase IgM-NAA especially if we understand how diverse infectious agents increase IgM-ALA. Studies are also needed to determine if prolonged high IgM-NAA levels can induce excess immunosuppression. One could also use enriched IgM intravenous preparations to acutely treat patients with uncontrolled inflammation. Cell therapy, especially with IgM pretreated DC, could provide an alternative approach requiring minimal quantities of IgM to prevent ischemic acute renal failure (e.g., in high-risk patients undergoing cardiac surgery) or delayed graft function after renal transplantation (4).

## Author Contributions

The author confirms being the sole contributor of this work and approved it for publication.

## Conflict of Interest Statement

The author declares that the research was conducted in the absence of any commercial or financial relationships that could be construed as a potential conflict of interest.
